# Strain Variation Can Significantly Modulate the miRNA Response to Zika Virus Infection

**DOI:** 10.3390/ijms242216216

**Published:** 2023-11-11

**Authors:** Suwipa Ramphan, Chanida Chumchanchira, Wannapa Sornjai, Thanathom Chailangkarn, Anan Jongkaewwattana, Wanchai Assavalapsakul, Duncan R. Smith

**Affiliations:** 1 Institute of Molecular Biosciences, Mahidol University, Nakhon Pathom 73170, Thailand; r.suwipa.earn@gmail.com (S.R.); wannapa.sor@mahidol.ac.th (W.S.); 2 Department of Biology, Faculty of Sciences, Chiang Mai University, Chiang Mai 50200, Thailand; cha.dreamii@gmail.com; 3 National Center for Genetic Engineering and Biotechnology, National Science and Technology Development Agency, Bangkok 12120, Thailand; thanathom.cha@biotec.or.th (T.C.); anan.jon@biotec.ot.th (A.J.); 4 Department of Microbiology, Faculty of Sciences, Chulalongkorn University, Bangkok 10330, Thailand; wanchai.a@chula.ac.th

**Keywords:** ZIKV, microRNA, miRNA, miR-34a, heat shock protein 70 kDa, HSP70

## Abstract

Zika virus (ZIKV) is a mosquito-transmitted virus that has emerged as a major public health concern due to its association with neurological disorders in humans, including microcephaly in fetuses. ZIKV infection has been shown to alter the miRNA profile in host cells, and these changes can contain elements that are proviral, while others can be antiviral in action. In this study, the expression of 22 miRNAs in human A549 cells infected with two different ZIKV isolates was investigated. All of the investigated miRNAs showed significant changes in expression at at least one time point examined. Markedly, 18 of the miRNAs examined showed statistically significant differences in expression between the two strains examined. Four miRNAs (miR-21, miR-34a, miR-128 and miR-155) were subsequently selected for further investigation. These four miRNAs were shown to modulate antiviral effects against ZIKV, as downregulation of their expression through anti-miRNA oligonucleotides resulted in increased virus production, whereas their overexpression through miRNA mimics reduced virus production. However, statistically significant changes were again seen when comparing the two strains investigated. Lastly, candidate targets of the miRNAs miR-34a and miR-128 were examined at the level of the mRNA and protein. HSP70 was identified as a target of miR-34a, but, again, the effects were strain type-specific. The two ZIKV strains used in this study differ by only nine amino acids, and the results highlight that consideration must be given to strain type variation when examining the roles of miRNAs in ZIKV, and probably other virus infections.

## 1. Introduction

Zika virus (ZIKV) is an arthropod-borne virus (arbovirus) in the genus *Flavivirus* that is transmitted primarily by *Aedes* spp. mosquitoes. ZIKV was first isolated from a sentinel rhesus monkey in Uganda in 1947 [1]. Following that first report, ZIKV was found to be wildly distributed through Africa and Asia and was characterized into two lineages, the African and Asian lineages [2,3,4]. Although widely distributed, there were few reports of human infection in the following 60 years [3]. In 2007 a small outbreak of Zika fever was reported in the Yap Islands of Micronesia [5], followed a few years later by a larger outbreak in French Polynesia [6]. The outbreak in French Polynesia marked the start of the worldwide spread of the Asian lineage of ZIKV [3], and as of February 2022 the World Health Organization has reported 89 countries and territories as having ZIKV transmission, and transmission is still ongoing [7].

Most cases of human infection with ZIKV are asymptomatic, but infection can result in a number of symptoms including fever, rash, headache and muscle and joint pain [8]. The symptoms are generally relatively self-limiting. In rare cases, more severe symptoms can occur, including the autoimmune-mediated Guillain–Barré syndrome [9] and, where a pregnant woman in her first or second trimester becomes infected, congenital Zika syndrome (CZS), in which the developing fetus can show severe developmental injury including microcephaly as a consequence of infection of the fetus [10].

ZIKV has a positive-sense single-stranded RNA genome that is translated to a single polyprotein containing three structural proteins (capsid: C, pre-membrane/membrane: prM/M, envelope: E) and seven non-structural (NS) proteins (NS1, NS2A, NS2B, NS3, NS4A, NS4B, NS5) [11]. The structural proteins form the new virion, while the non-structural proteins form a complex that replicates the genomic material through a negative sense intermediate [12], as well as aiding in reprograming the host cell machinery to facilitate virus replication [13] and aid in blunting the host cell innate immune response [14].

microRNAs (miRNAs) are non-coding RNAs that regulate gene expression at the post-transcriptional level through the formation of an RNA-induced silencing complex (RISC) in a process known as RNA interference (RNAi) [15]. miRNAs silence target mRNAs by the binding of 6–8 nucleotides (known as the seed region) of the miRNA to the 3′UTR of a cognate mRNA, resulting in translation repression or mRNA degradation [16]. Studies have shown that miRNAs are involved in many cellular processes, including differentiation [17], proliferation [18], and apoptosis [19]. Moreover, miRNAs have been reported to play roles in virus infection as a host cell defensive process. For example, Let-7c inhibits dengue virus (DENV) replication in human hepatoma cells through modulation of the cellular oxidative response [20]; similarly, miR-223 inhibits DENV by negatively regulating microtubule proteins in human umbilical vein cells [21], while the miR-34 family inhibits flavivirus replication through the repression of the Wnt signaling pathway in Hela cells [22]. In contrast, some miRNAs are essential for promoting viral replication during infection, such as miR-21, which promotes DENV replication in human hepatoma cells [23]. In the case of ZIKV infection, the microcephalic defect has been reported to be regulated by miR-9 during virus infection in mice [24].

However, the mechanism of ZIKV-induced microcephaly and miRNA regulation during ZIKV infection remains poorly understood. As noted earlier, the ZIKV outbreaks in Micronesia and French Polynesia and the subsequent worldwide spread were caused by Asian-lineage ZIKV [25]. To further understand the interplay between miRNAs and ZIKV, two ZIKV Asian-lineage isolates were used in this study, one of which was isolated from a Zika fever patient [26] while the second was isolated from a case of ZIKV-associated microcephaly [27]. Both viruses were circulating in Thailand between 2012 and 2017 [26,27]. An initial screening of candidate miRNAs was undertaken in human adenocarcinomic alveolar basal epithelial A549 cells [28], and four candidates (miR-21, miR-34a, miR-128 and miR-155) were selected for further validation, both in A549 cells and in neural progenitor cells (NPCs) generated from induced pluripotent stem cells (iPSCs). Collectively, our results identify miR-34a as a mediator of ZIKV infection through its effects on the expression of heat shock protein 70.

## 2. Results

### 2.1. Validation of miRNA Expression Level in ZIKV-Infected Cells

To investigate the regulation of miRNAs in ZIKV infection, a total of 50 candidate miRNAs (Supplemental Table S1) were selected based on their reported involvement in the infection of other viruses, and whether they were expressed in A549 cells was determined by RT-PCR. The results showed that 22 of the miRNAs were detectable in A549 cells (Supplemental Table S5). To determine the effect of ZIKV infection on the expression of the remaining 22 miRNA candidates (hsa-miR-10b-5p, hsa-miR-15b-5p, hsa-miR-16-3p, hsa-mir-106b-5p, hsa-mir-125a-5p, hsa-miR-128-3p, hsa-miR-155-5p, hsa-miR-192-5p, hsa-miR-21-5p, hsa-miR-215-5p, hsa-miR-218-5p, hsa-miR-23b-3p, hsa-miR-27a-3p, hsa-miR-29b-3p, hsa-miR-30a-3p, hsa-miR-30b-5p, hsa-miR-30e-5p, hsa-miR-34a-5p, hsa-miR-424-5p, hsa-miR-497-5p, hsa-miR-532-5p, Let-7a-5p), A549 cells were separately infected with one of two strains of ZIKV (SV0010/15 or MU1-2017) at MOI 2 or mock-infected, and cells were collected at 6, 12, 24 and 48 h post-infection. In parallel, the level of infection for each virus was determined by flow cytometry and the results (Supplemental Figure S1) showed approximately equal levels of infection at each time point. To determine the effect of ZIKV infection on miRNA expression, cells were collected, followed by RNA extraction, cDNA synthesis and quantitative reverse-transcription PCR (qPCR). The qPCR was performed using specific primers, with the small nuclear U_6_ RNA used as an internal control (specific primers are shown in Supplemental Table S1).

**Figure 1:**
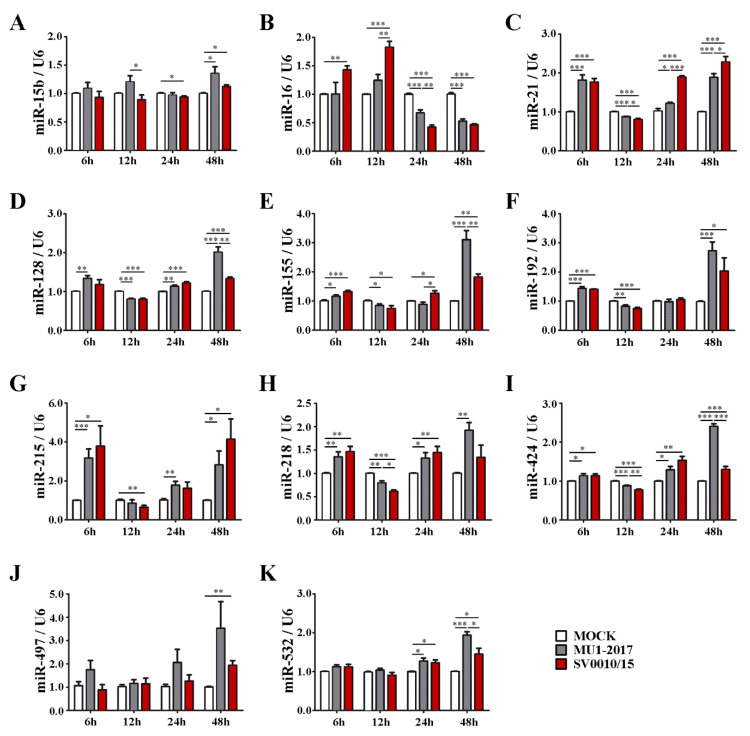
miRNA (miR-15b, miR-16, miR-21, miR-128, miR-155, miR-192, miR-215, miR-218, miR-424, miR-497, miR-532) expression levels after infection with one of two strains of ZIKV. A549 cells were infected with ZIKV MU1-2017 or ZIKV SV0010/15 at MOI 2 for 6, 12, 24 and 48 h. (**A**–**K**) Levels of expression of each miRNA were quantified by RT-qPCR using specific primers with a 2^−∆∆CT^ calculation method and a small nuclear RNA; U6 was used as an internal control. All experiments were undertaken independently as biological triplicates; error bars represent standard error of mean. *p* value * < 0.05, ** < 0.01 and *** < 0.001.

The results of the RT-qPCR (Figure 1 and Figure S2) showed that all miRNAs analyzed showed some alteration of expression at one or more time points examined. Several common expression patterns were observed. Several miRNAs showed alterations in expression for at least one of the two ZIKVs at all time points, including miR-16, miR-21, miR-30b, miR-125a, miR-128, miR-155, miR-218 (Figure 1B–E,H,I), as well as miR-10b, miR-125a, miR-23b, miR-30a, miR-30b (Supplemental Figure S2B–D,G,H). While the general trend for these miRNAs was an increase in expression, several miRNAs showed significantly reduced expression at the 12 or 24 h time points, such as miR-21, miR-128, miR-218, miR-23b, miR-30a, miR-30b (Figure 1C,D,H and Figure S2D,G,H), in an expression pattern that shows early upregulation, then downregulation, then late upregulation. Some miRNAs showed only changes in expression at late time points (24 to 48 h post-infection), including miR-15b, miR-497, miR-532 and miR-106b (Figure 1A,J,K and Figure S2A). Another pattern of expression observed included early (6 and 12 h post-infection) upregulation followed by late downregulation, such as with miR-16 (Figure 1B).

There were a number of statistically significant differences in expression levels of miRNAs when comparing between the two viruses. In fact, only miR-192, miR-215 and miR-497 (Figure 1F,G,J) and miR-106b (Supplemental Figure S2A) showed no differences in miR expression levels at all time points examined. Significant differences in expression levels were commonly found at the last time point examined (48 h post-infection) as was seen for miR-21, miR-128, miR-155, miR-424, miR-532, miR-125a, miR-27a, miR-29b, miR-30a, miR-30b, miR-30e, miR-34a (Figure 1C–E,I,K and Figure S2C,E–J). Only two miRs, miR-29b and miR-30e (Supplemental Figure S2F,I), showed differential expression levels at the earliest time point examined (6 h post-infection).

### 2.2. Inhibition of miRNA Expression

**Figure 2:**
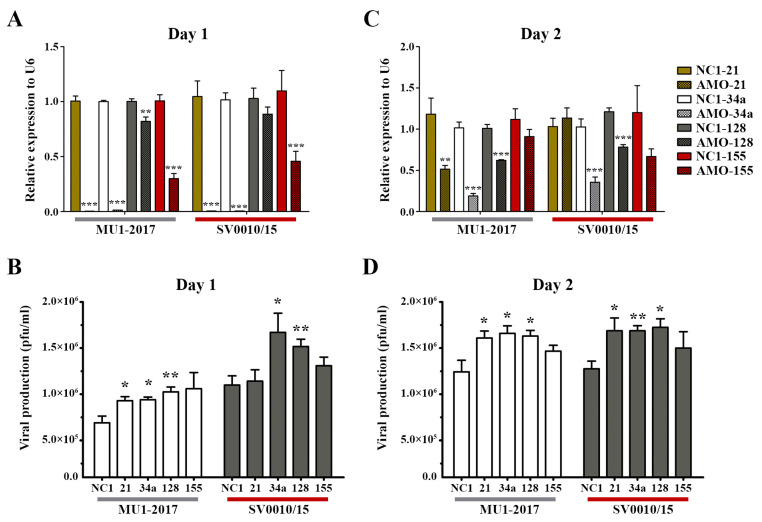
Inhibition of miRNA in ZIKV-infected cells. A549 cells were infected with ZIKV MU1-2017 or ZIKV SV0010/15 followed by transfection with each AMO. AMO-NC1 was used as a scrambled control. On days 1 to 2 post-infection, cells were collected and RNA extracted to determine the miRNA expression levels and the supernatant was collected to determine viral production. Relative miRNA expression level in AMO-treated cells compared to the scramble control was determined by RT-qPCR with U6 used as an internal control (**A**,**C**). Viral production after AMOs treatment was determined by plaque assay (**B**,**D**). Experiments were undertaken independently in biological triplicate with technical duplicate plaque assay and technical triplicate RT-qPCR; error bars represent standard error of mean. *p* value * < 0.05, ** < 0.01 and *** < 0.001.

To clarify the role of miRNAs in ZIKV infection, four miRNAs (hsa-miR-128-3p, hsa-miR-155-5p, hsa-miR-21-5p, hsa-miR-34a-5p) were selected for further investigation based upon their reported involvement in infection by other viruses [22,23,29,30,31,32,33]. A549 cells were either mock- or ZIKV-infected at MOI 2 for 2 h followed by transfection with 100 nM of anti-miRNA oligonucleotides (AMO) directed to the four selected miRNAs. Post-infection transfection was selected as it gave a greater degree of inhibition than transfection followed by infection (Supplemental Figure S3). On days 1–2 post-transfection, the supernatant and cells were collected for determination of viral titer and miRNA level, respectively. The results for MU1-2017 infection showed that on day 1 post-transfection, AMO treatment significantly reduced miRNA expression for all transfections (Figure 2A). An increase in viral titer was seen for miR-21, miR-34a and miR-128, but, although virus titer was increased after AMO-155 treatment, it was not statistically significant (Figure 2B). Similar results were seen in the ZIKV SV0010/15 infection, with AMO treatment reducing miRNA expression in all transfections, although the reduction seen with AMO-128 treatment was not significant. Virus titer was significantly increased after AMO-34a and AMO-128 treatment and increased, but not significantly, after AMO-21 and AMO-155 treatment (Figure 2A,B).

On day 2 post-transfection in the MU1-2017 infection, miRNA expression remained significantly reduced after AMO-21, AMO-34a and AMO-128 treatments, and this was matched by a concordant increase in viral titer for these transfections (Figure 2C,D). In the AMO-155 treatment, miR-155 expression was reduced, but not significantly, and, similarly, virus titer was not significantly different from the scrambled control (Figure 2C,D). In the SV0010/15 infection, miR-34a and miR-128 expression was reduced, and virus titer was significantly increased, while miR-21 expression was similar to that seen with the scrambled control, but, interestingly, virus titer was still significantly increased (Figure 2C,D). miR-155 expression was reduced, but not significantly, and virus titer was also increased, but not significantly (Figure 2C,D).

To show the effect of the various AMOs in the absence of infection, the expression of the four selected miRNAs was investigated in mock-infected cells. The results (Supplemental Figure S4) showed significant downregulation of the miRNAs after AMO treatment as compared to scrambled control-treated cells, particularly on day 1 post-transfection (Supplemental Figure S4). Surprisingly, however, treatment with AMO-21 resulted in an increase in miR-21 on day 2 post-mock infection (Supplemental Figure S4). While the day 1 results are clear and consistent, because of the apparent inconsistency on day 2, this miR was not selected for further investigation.

### 2.3. Treatment with Mimic miRNAs

**Figure 3:**
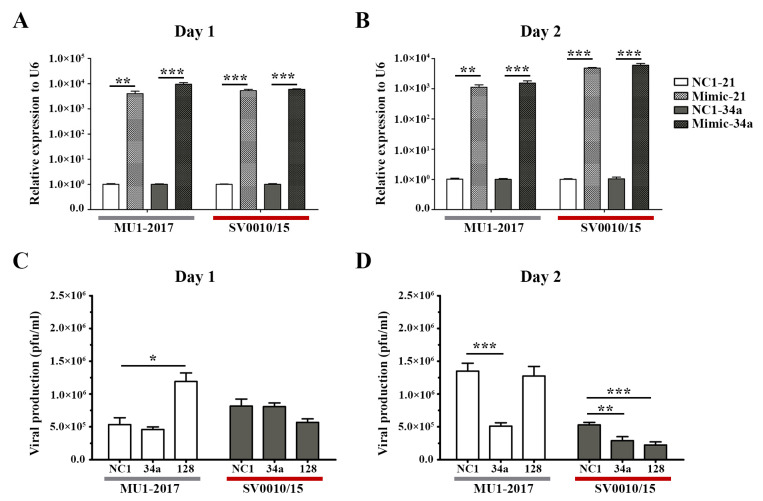
Overexpression of miRNAs in ZIKV-infected cells. Mimic miRNAs (mimic-34a and mimic-128) were reverse-transfected into cells followed by infection with ZIKV- MU1-2017 or ZIKV SV0010/15 as appropriate. Mimic-NC1 was used as a scrambled control. RNA from cells and supernatant were collected to determine the miRNAs’ expression levels and viral production at the indicated time points. (**A**,**B**) Relative miRNA expression level in mimic miRNA-treated cells compared to the scramble control, with U6 used as an internal control. (**C**,**D**) Viral production after mimic miRNA treatment was determined by plaque assay. Experiments were undertaken independently as biological triplicates with technical duplicate plaque assay and technical triplicate RT-qPCR; error bars represent standard error of mean. *p* value * < 0.05, ** < 0.01 and *** < 0.001.

As reduced expression of miRNAs resulted in increased virus production, logic would suggest that increasing the level of miRNAs would reduce virus titer. To explore this, mimic miRNAs were obtained for two of the miRNAs analyzed in the previous section, namely miR-34a and miR-128. A549 cells were therefore either mock-transfected or mimic miRNAs-transfected for 24 h followed by infection with each strain of ZIKV at MOI 2. On days 1 and 2 post-infection, supernatant and cells were collected for determination of viral titer and mimic/miRNA levels, respectively. Expression of both mimic/miRNAs was significantly increased on both days for both virus infections (Figure 3A,B). On day 1 post-infection, the only significant change in virus titer was seen after mimic-128 treatment in the MU1-2017 infection, but, surprisingly, an increase in virus titer was observed (Figure 3C). The other mimic treatment showed small, but not significant, reductions in virus titer. On day 2 post-infection, miR-34a mimic treatment resulted in a significant reduction in virus titer for both viruses (Figure 3D), and a significant reduction was seen for miR-128 mimic treatment in ZIKV SV0010/15 infection (Figure 3B). In the MU1-2017 infection, a slight but non-significant reduction in virus production was observed after miR-128 mimic treatment. With the exception of mimic miR-128 treatment on day 1 post-infection for MU1-2017, mimic treatment was broadly antiviral, and this was consistent with the previous results seen with downregulation of miRNA expression.

To show the effects of the various mimics in the absence of infection, the expression of the two selected miRNAs was investigated in mock-infected cells. The results (Supplemental Figure S5) showed significant upregulation of the miRNAs after mimic treatment as compared to scrambled control-treated cells, on both days investigated (Supplemental Figure S5).

### 2.4. Predicted Targets of miR-34a and miR-128

To predict the target mRNAs of miR-34a and miR-128, the miRbase [34], TargetScanHuman 7.1 [35] and miRDB [36] miRNA target prediction tools were used. The predicted mRNA target binding sites are shown in Table 1. Aldolase A, fructose bisphosphate (ALDOA), heat shock 70 kDa protein 1B (HSPA1B, HSP70) and notch 2 (NOTCH2) were predicted as targets of miR-34a, while Musashi RNA-binding protein 2 (MSI2), prohibitin (PHB1) and ubiquitin-conjugating enzyme E2 E2 (UBE2E2) were predicted as targets of miR-128. These targets were then investigated in ZIKV-infected cells. Therefore, A549 cells were either mock-infected or infected with each strain of ZIKV at MOI 2. Cells were collected to determine target mRNAs and protein expression levels. On day 1 post-infection, the analysis of target mRNAs showed that ALDOA showed a slight increase in mRNA expression in MU1-2017 infection, and a significant increase in mRNA expression in SV0010/15 infection (Supplemental Figure S6A). However, by day 2 post-infection, discordant results were seen in that ALDOA mRNA was significantly decreased in MU1-2017 and significantly increased in SV0010/15 infection as compared to mock, and expression levels between the two virus infections were also significantly different (Supplemental Figure S6A).

HSP70 mRNA showed significantly differential expression between the two ZIKV strains on both days investigated (Supplemental Figure S6B). On day 1 post-infection, only in SV0010/15 infection was HSP70 mRNA significantly increased as compared to mock, while on day 2 post-infection, both virus infections showed significant increases over mock (Supplemental Figure S6B).

On day 1 post-infection, the mRNA for NOTCH2 was marginally but not significantly increased in both MU1-2017 and SV0010/15 infections as compared to mock, and by day 2 post-infection, both virus infections showed a significant increase in NOTCH2 expression over mock, as well as a differential level of expression between the two virus infections, with the SV0010/15 infection showing the higher level of NOTCH2 expression (Supplemental Figure S6C).

MSI2 mRNA was significantly downregulated on day 1 post-infection in MU1-2017 infection, but not in SV0010/15 infection (Supplemental Figure S6D), but it was significantly downregulated in both virus infections on day 2 post-infection (Supplemental Figure S6D).

PHB1 mRNA was slightly, but significantly, upregulated on day 1 post-infection in SV0010/15 infection, but no significant difference was seen for either virus on day 2 post-infection, although we note some greater variability in expression between replicates for PHB1 mRNA in SV0010/15 infection on day 2 post-infection (Supplemental Figure S6E). Lastly, UBE2E2 mRNA was differentially expressed between the two virus infections on day 1 post-infection, although in neither infection was a significant difference seen as compared to mock infection (Supplemental Figure S6F). By day two post-infection, both virus infections showed significantly increased expression of UBE2E2 mRNA as compared to mock, with no significant difference seen between the two virus infections (Supplemental Figure S6F).

### 2.5. Effect of miR-34a or miR-128 Inhibition on the Predicted Targets

To confirm the regulation of miR-34a and miR-128 in ZIKV infection, miR-34a or miR-128 expression was inhibited. A549 cells were either mock-infected or infected with each strain of ZIKV at MOI 2 and transfected with AMO-34a or AMO-128. At 1 and 2 days post-transfection, cells were collected for examination of mRNA expression level and protein expression levels. On day 1 post-transfection with AMO-34a, there was no significant change in ALDOA mRNA expression shown, while on day 2 post-transfection, the expression of ALDOA mRNA was significantly increased in MUI-2017 infection as compared to both mock and SV0010/15 infection (Supplemental Figure S7A). A largely similar pattern was seen with HSP70, with no significant change on day 1 post-transfection (Supplemental Figure S7B), while on day 2 post-transfection, a significant increase in HSP70 mRNA expression was seen in both MU1-2017 and SV0010/15 infections as compared to mock, and, moreover, a significant difference was seen between expression levels of HSP70 mRNA when comparing between MU1-2017 and SV0010/15 infections. Markedly, HSP70 expression in MU1-2017 infection on day 2 post-infection was significantly increased over the cognate scrambled miRNA control (Supplemental Figure S7B).

NOTCH2 expression was significantly reduced on day 1 post-transfection in SV0010/15 infection compared to both mock infection and MU1-2017 infection, but by day 2, the transfection expression of NOTCH2 was significantly increased in MU1-2017 infection as compared to both mock infection and SV0010/15 infection (Supplemental Figure S7C). Similarly, a significant difference was seen on day 2 post-transfection when comparing expression levels of NOTCH2 between SV0010/15 and mock infection (Supplemental Figure S7C). Interestingly, NOTCH2 showed significantly reduced expression on day 2 post-transfection in MU1-2017 as compared to the cognate scrambled miRNA control (Supplemental Figure S7C).

For the targets of miR-128, the expression of MSI2 mRNA was not significantly changed on day 1 post-transfection as a consequence of infection, but on day 2, transfection expression of MSI2 mRNA was significantly decreased in SV0010/15 infection, but not in MU1-2017 infection as compared to mock (Supplemental Figure S7D). However, a significant difference in MSI2 mRNA expression was seen between the mock miR-128 transfection and the cognate scrambled control (Supplemental Figure S7D).

PHB mRNA expression was increased on day 1 post-transfection in SV0010/15 infection as compared to mock infection, while PHB mRNA expression was not significantly altered in MU1-2017 infection. On day 2, transfection of PHB mRNA expression was significantly increased in both MU1-2017 and SV0010/15 infection as compared to mock infection (Supplemental Figure S7E). Significant differences were observed between the miR-128 transfections and the scrambled miRNA controls for SV0010/15 on day 1 post-transfection, and for both MU1-2017 and SV0010/15 on day 2 post-transfection (Supplemental Figure S7E). Lastly, expression of UBE2E2 mRNA was significantly increased in SV0010/15 infection as compared to mock infection on day 1 post-transfection, while on day 2 post-transfection, UBE2E2 expression was increased in SV0010/15 as compared to both mock infection and MU1-2017 infection (Supplemental Figure S7E).

**Figure 4:**
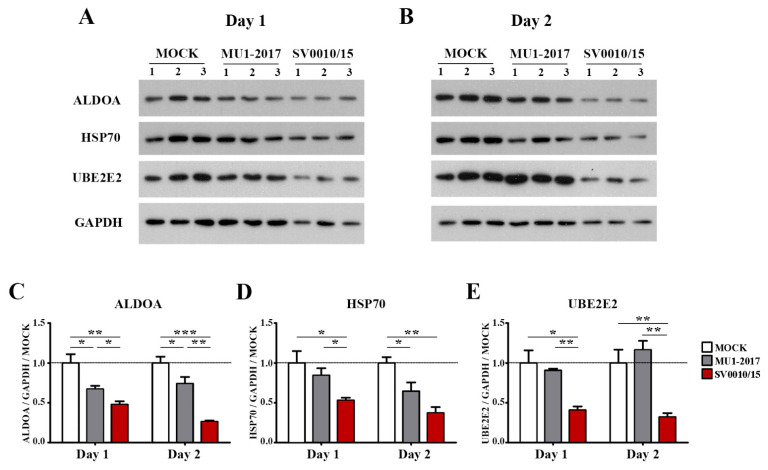
Expression of putative target proteins of miR-34a and miR-128 in ZIKV infection. Cells were infected with ZIKV MU1-2017 or ZIKV SV0010/15 for 1 (**A**,**C**–**E**) or 2 days (**B**–**E**), after which cells were collected, followed by determination of target proteins on (**A**) day 1 and (**B**) day 2. Bar graph presents the relative protein expression level of (**C**) Aldolase A (ALDOA), (**D**) heat shock protein 70 (HSP70) and (**E**) ubiquitin-conjugating enzyme E2E 2 (UBE2E2). GAPDH was used as an internal. Experiments were undertaken independently in biological triplicate. Error bars represent standard error of mean. *p* value * < 0.05, ** < 0.01 and *** < 0.001. Cropped images of Western blots are shown, and full un-cropped images can be found in the Supplementary Materials.

The protein expression of the miRNA-34a regulated genes ALDOA and HSP70, the miRNA-128 regulated gene UBE2E2 and an internal control (GAPDH) was investigated by Western blot analysis (Figure 4A,B), with quantitation as shown in Figure 4C–E. The results showed that the expression of ALDOA was reduced in both MU1-2017 and SV0010/15 infections as compared to mock infection on both days 1 and 2 post-infection, and, moreover, on both days, expression of ALDOA was significantly reduced in SV0010/15 infection as compared to MU1-2017 infection (Figure 4A–C). HSP70 expression was observed to be significantly reduced on day 1 post-infection in SV0010/15 as compared to both mock infection and MU1-2017 infection (Figure 4A,D), and expression of HSP70 was significantly reduced as compared to mock infection for both SV0010/15 and MU1-2017 infections on day 2 post-infection (Figure 4B,D). The expression of UBE2E2 was significantly reduced in SV0010/15 infection on day 1 and 2 post-infection compared to mock infection and MU1-2017 infection (Figure 4A,B,E).

### 2.6. Effects of AMO-34a and AMO-128 on Target Protein Expression during Infection

**Figure 5:**
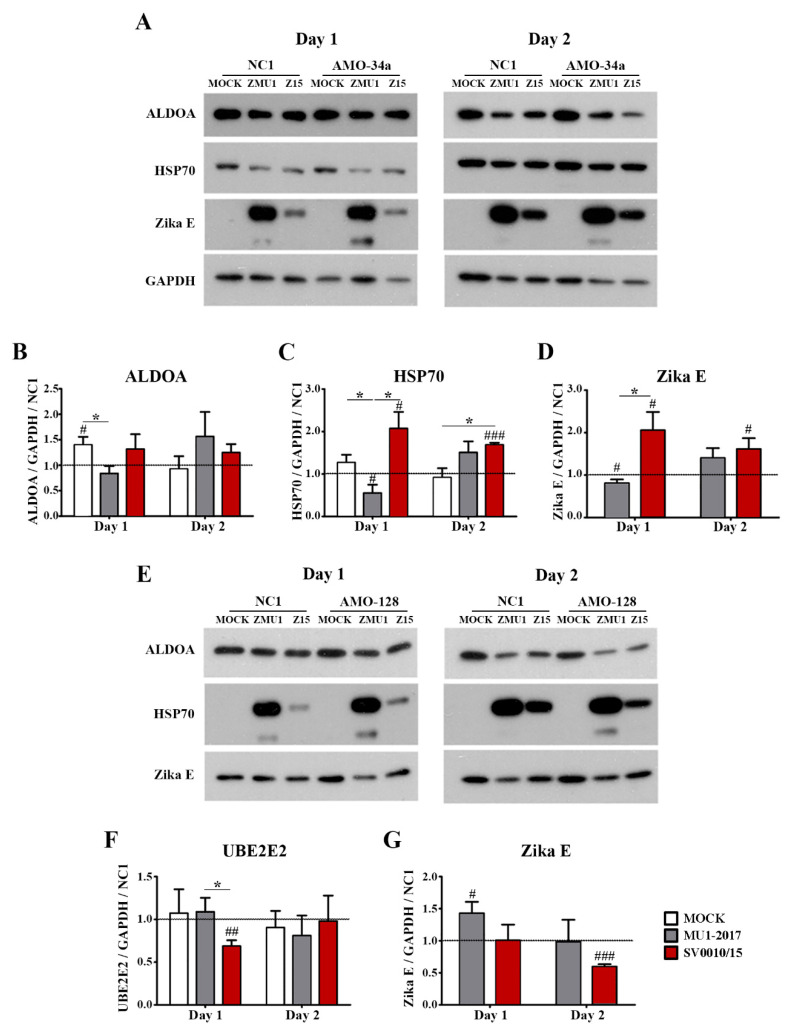
Effects of miRNA inhibition in ZIKV-infected cells. A549 cells were infected with ZIKV MU1-2017 or ZIKV SV0010/15 followed by transfection with AMO-34a or AMO-128. AMO-NC1 was used as a scrambled control. Cells were collected on days 1 and 2 post-infection, followed by determination of target protein expression for (**A**) ALDOA, HSP70 and Zika E protein expression in AMO-34a treated cells and (**E**) expression of UBE2E in AMO-128 treated cells. Bar graphs present relative protein expression levels of (**B**–**D**) ALDOA, HSP70 and Zika E and (**F**,**G**) UBE2E2 and Zika E protein. GAPDH was used as an internal control and experiments were undertaken independently as biological triplicates, while error bars represent the standard error of mean. *p* value * < 0.05, # *p* < 0.05, ## *p* < 0.01, ### *p* < 0.001 when comparing between sample and scrambled control. Cropped images of western blots are shown, and full un-cropped images can be found in the Supplementary Materials.

To look at the effects of the inhibition of miR-34a and miR-128 during virus infection, cells were transfected with AMO-34a or AMO-128 as appropriate, and subsequently were mock-infected, or infected with ZIKV SV0010/15 or MU1-2017 as appropriate. Target protein expression (ALDOA and HSP70 for inhibition of miR-34a and UBE2E2 for inhibition of miR-128 expression) was examined by Western blot, together with determining the expression of ZIKV E protein and GAPDH as an internal control. Results (Figure 5) showed that ALDOA expression was significantly reduced in ZIKV MU1-2017 infection on day 1 post-infection (Figure 5A,B) but that no significant changes in expression levels were seen in either ZIKV SV0010/15 or ZIKV MU102017 infections on day 2 post-infection (Figure 5A,B). In contrast, HSP70 expression was found to be significantly increased as compared to mock in SV0010/15 infection on both days 1 and 2 post-infection, and significantly increased as compared to MU1-2017 on day 1 post-infection, but not on day 2 post-infection (Figure 5A,C). On day 1 post-infection, ZIKV E protein expression was increased as compared to the scrambled control in the SV0010/15 infection, and reduced as compared to the scrambled control in the MU1-2017 infection, and levels of ZIKV E protein were significantly different between the two ZIKV strains (Figure 5A,D). By day 2 post-infection, levels of ZIKV E protein were significantly increased as compared to the scrambled control in SV0010/15 infection, but not in MU1-2017 infection (Figure 5A,D).

Transfection of cells with AMO-128 to reduce miRNA-128 levels followed by infection with ZIKV-MU1-2017 or ZIKV SV0010/15 significantly reduced levels of UBE2E2 on day 1 post-infection in SV0010/15 infection as compared to MU1-2017 infection and when compared to the scrambled control (Figure 5E,F), but no differences in expression were seen on day 2 post-infection (Figure 5E,F). ZIKV E protein levels were significantly increased as compared to the scrambled control in MU1-2017 infection on day 1 post-infection and were significantly reduced as compared to the scrambled control in SV0010/15 infection on day 2 post-infection (Figure 5E,G).

### 2.7. Effect of Mimic miR-34a and miR-128 on Predicted Targets

**Figure 6:**
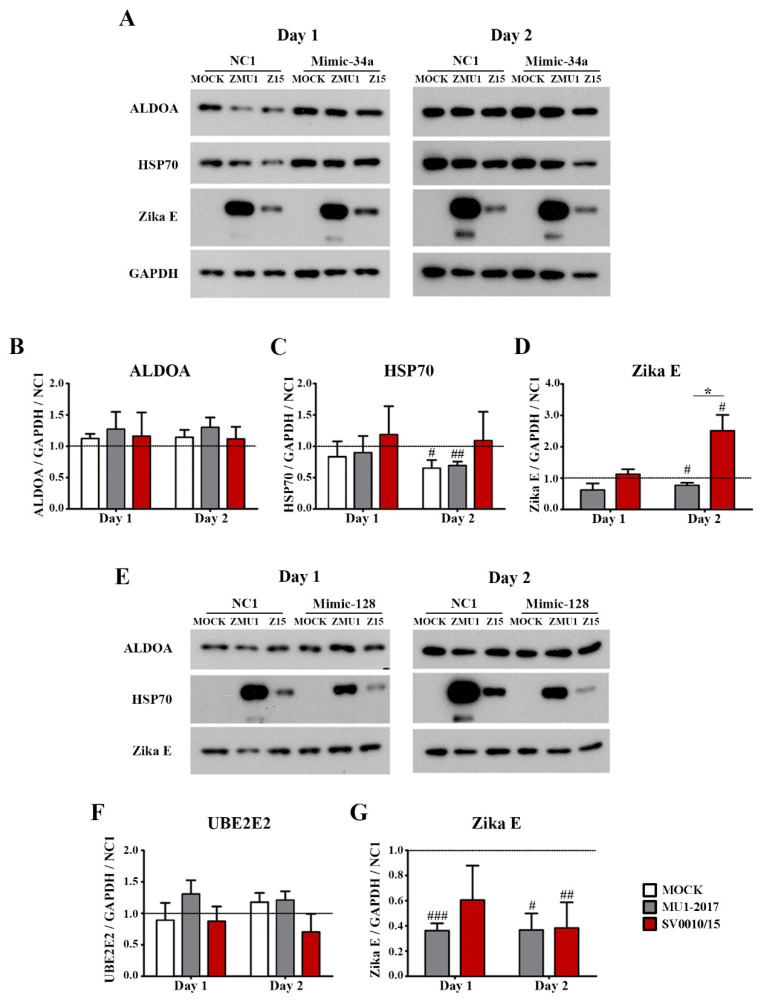
Effects of mimic miRNA expression in ZIKV-infected cells. A549 cells were treated with mimic-34a or mimic-128 and mimic-NC1 was used as a scramble control followed by infection with ZIKV MU1-2017 or ZIKV SV0010/15. Cells were collected followed by determination of target protein expression of (**A**) ALDOA, HSP70 and Zika E protein expression in mimic-34a-treated cells. Bar graphs present relative protein expression levels of (**B**–**D**) ALDOA, HSP70 and Zika E. (**E**) UBE2E2 and Zika E protein expression in mimic-128-treated cells. Bar graphs present relative protein expression level of (**F**,**G**) UBE2E2 and Zika E. GAPDH was used as an internal control. Experiments were undertaken independently in biological triplicate. Error bars represent the standard error of mean. *p* value * < 0.05, # *p* < 0.05, ## *p* < 0.01, ### *p* < 0.001 when comparing between sample and scrambled control. Cropped images of western blots are shown, and full un-cropped images can be found in the Supplementary Materials.

To determine the effect of increased miR levels on predicted target protein expression, two mimics (mimic-miR-34a and mimic-miR-128) were generated. Subsequently, A549 cells were either mock-transfected or mimic miRNA- (or a scrambled control) transfected for 24 h followed by infection with each strain of ZIKV at MOI 2. Cells were collected on days 1 and 2 post-infection to determine mRNA expression levels for the putative targets of the two miRNAs, namely ALDOA, HSP70 and NOTCH2 for miR-34a and MSI2, PHB and UBE2E2 for miR-128 (Supplemental Figure S8). In addition, protein levels of selected targets (namely ALDOA, HSP70 as putative targets of miR-34a and UBE2E2 as a putative target of miR-128) were established by Western blot analysis in parallel with establishing levels of ZIKV E protein and GAPDH as a control (Figure 6).

Results showed that mimic-34a treatment resulted in a significant increase in expression of ALDOA mRNA as compared to mock infection for both strains of ZIKV on day 1 post-infection (Supplemental Figure S8A), and, additionally, ALDOA expression was significantly higher in SV0010/15 infection than in ZIKV MU1-2017 infection (Supplemental Figure S8A). On day two post-infection, only ZIKV MU1-2017 infection showed increased expression of ALDOA as compared to mock infection (Supplemental Figure S8A). Markedly, all transfections showed a significant increase in expression of ALDOA as compared to the scrambled control in both mock infection and ZIKV infections and on both days post-infection (Supplemental Figure S8A).

HSP70 mRNA was significantly reduced as compared to the scrambled control for all transfections (Supplemental Figure S8B), including the mock infection. On day 1 post-infection, HSP70 mRNA expression in the mock infection was significantly lower than both ZIKV infections, and the level of HSP70 mRNA in the SV0010/15 infection was significantly higher than the level in the MU1-2017 infection (Supplemental Figure S8B). On day 2 post-infection, levels of HSP70 mRNA remained significantly below the level in the scrambled control, and no differences in expression level were seen between ZIKV infections and mock infection (Supplemental Figure S8B).

NOTCH2 mRNA expression in SV0010/15 infection was significantly increased as compared to mock transfection and MU1-2017 infection on day 1 post-infection (Supplemental Figure S8C), and the expression of NOTCH2 RNA was significantly reduced in MU1-2017 infection as compared to the scrambled control (Supplemental Figure S8C). On day 2 post-infection, the expression of NOTCH2 was significantly increased in the mock infection as compared to both ZIKV strain infections, and was additionally significantly increased as compared to the scrambled control (Supplemental Figure S8C).

For the mimic-128-treated samples, MSI2 expression on day 1 post-infection was significantly increased in the MU1-2017 infection as compared to both mock infection and SV0010/15 infection, and MSI2 expression in SV0010/15 infection was significantly reduced as compared to the scrambled control (Supplemental Figure S8D). In a similar manner, MSI2 expression on day 2 post-infection was significantly increased as compared to the levels in both mock infection and SV0010/15 infection, and moreover, levels of MSI2 mRNA expression in both SV0010/15 infection and MU1-2017 infection were significantly increased as compared to the scrambled control (Supplemental Figure S8D).

The expression of PHB mRNA on day 1 post-infection was significantly reduced as compared to the scrambled control in both mock infection and ZIKV infections (Supplemental Figure S8E), and the level of PHB expression was reduced in the mock infection as compared to both ZIKV infections (Supplemental Figure S8E). On day 2 post-infection, levels of PHB mRNA were reduced in the mock infection compared to the scrambled control and both ZIKV infections (Supplemental Figure S8E). Lastly, the expression of UBE2E2 mRNA was significantly reduced on day 1 post-infection in both mock-infected and ZIKV-infected samples as compared to the scrambled control, and levels in the mock infection were significantly reduced as compared to both ZIKV infections (Supplemental Figure S8F). On day 2 post-infection, UBE2E2 mRNA expression was significantly different from the scrambled transfection for both mock and ZIKV infections, although, while mock infection and SV0010/15 infection were reduced in UBE2E2 expression as compared to the scrambled control, MU1-2017 was significantly increased as compared to the scrambled control and levels were additionally significantly increased with reference to the levels detected in both mock infection and SV0010/15 infection (Supplemental Figure S8F).

In terms of protein expression after mimic treatment, ALDOA showed no change in expression level for both viruses on both days investigated (Figure 6A,B). For HSP70, no significant changes were seen on day 1 post-infection. On day 2 post-infection, HSP70 expression levels were significantly reduced as compared to the scrambled control for mock infection and MU1-2017 infection, but no significant difference was seen from the scrambled control for SV0010/15, and no significant difference was observed between either virus and mock, or between viruses (Figure 6A,C). No differences were observed for E protein expression on day 1 post-infection for cells treated with mimic-34a, but both ZIKV MU1-2017 and ZIKV SV0010/15 showed significant differences from the scrambled control on day 2 post-infection, although MU1-2017 was reduced as compared to the scrambled control and SV0010/15 was increased as compared to the scrambled control. In addition, a significant difference was seen in E protein expression between cells infected with MU1-2017 and SV0010/15 (Figure 6A,D).

The expression of UBE2E was examined for cells treated with mimic-128. No change in UBE2E2 expression was seen on either day post-infection and for all treatment conditions (mock, MU1-2017-infected, SV0010/15-infected) and time points (Figure 6E,F). The expression of E protein was reduced as compared to the scrambled control for MU1-2017 infection on day 1 post-infection, while the level of E protein was reduced as compared to the scrambled control for SV0010/15 infection, albeit not significantly (Figure 6E,G). However, E protein levels were significantly reduced for both MU1-2017 and SV0010/15 as compared to the scrambled control (Figure 6E,G). Putative target proteins were selected based on prior reports of their involvement in viral infections.

### 2.8. Determination of miRNA Expression Levels in ZIKV-Infected NPCs

**Figure 7:**
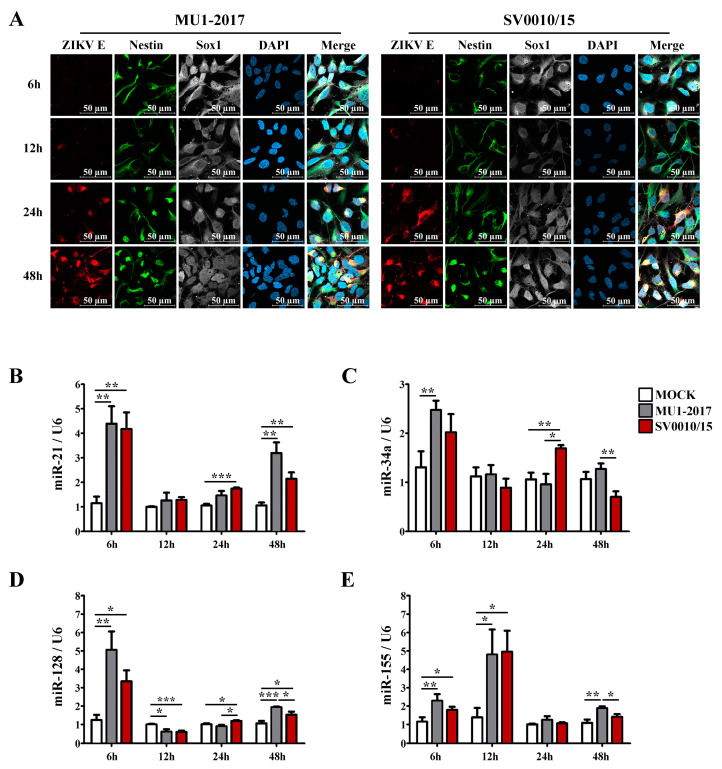
miRNA expression levels in ZIKV-infected neural progenitor cells (NPCs). (**A**) NPCs were infected with ZIKV MU1-2017 or ZIKV SV0010/15 for 6, 12, 24 and 48 h. After this, cells were incubated with primary antibodies to ZIKV E protein (red), nestin (green) and sox1 (white), followed by counter staining with DAPI (blue). In parallel, cells under the same conditions were used to extract RNA, and the relative expression of miRNA-21 (**B**), miRNA-34a (**C**), miRNA-128 (**D**) and miR-155 (**E**) were determined by RT-qPCR with normalization against U6 as an internal control. Experiments were undertaken as independent biological triplicates with technical triplicate RT-qPCR; error bars represent the standard error of mean. *p* value * < 0.05, ** < 0.01 and *** < 0.001.

To investigate the expression of selected miRNAs in a more biologically relevant system, human neural progenitor cells (NPCs) were generated from iPSCs, and their identity was confirmed by appropriate marker staining (Supplemental Figure S9). The 22 miRNAs that had generated miRNA bands in A549 cells (Supplementary Table S5) were rescreened using RNA from the newly generated NPC. A total of 12 miRNAs gave amplification products (Supplementary Table S6), which included the 4 miRNAs (miR-21, miR-34a, miRNA-128 and miR-155) investigated in A549 cells, which were again selected for further analysis. After that, NPCs were either mock-infected or infected with each strain of ZIKV at MOI 20 for 6, 12, 24 and 48 h, after which cells were either fixed for immunofluorescence assay or collected for RT-qPCR examination. Cells were stained for the NPC markers nestin and sox1 together with ZIKV E protein and were counterstained with DAPI. ZIKV E protein was first detected at 12 hpi for both virus strains, and the intensity of staining increased over the time of the experiment (Figure 7A). Results for the mock infection undertaken in parallel are shown in Supplemental Figure S10. The expression levels of the selected miRNAs showed that all of miR-21, miR-34a, miR-128 and miR-155 were upregulated at the early stage (6 hpi) of infection for both strains of ZIKV (Figure 7B–E), although miR-34a in SV0010/15 did not reach statistical significance (Figure 7C). For miR-21, miR-128 and miR-34a, the level of expression at 12 h was greatly reduced as compared to the 6 hpi time point, and levels were approximately equal to or lower than the mock control (Figure 7B–D). The exception was miR-155 (Figure 7E), which showed significantly increased expression as compared to mock. At 24 hpi, miR-21, -34a and -128 were significantly re-upregulated only in ZIKV SV0010/15 infection, and no expression level change was seen with either virus for miR-155 (Figure 7B–E). At 48 hpi, miR-21 and -128 were significantly upregulated by both strains of ZIKV (Figure 7B,D), while miR-34a and miR-155 were significantly upregulated in ZIKV MU1-2017 infection but not in ZIKV SV0010/15 infection (Figure 7C,E). Finally, significant differences in expression levels of the miRNAs when comparing between virus stains were seen at the latter time points for miR-34a (Figure 7C), miR-128 (Figure 7D) and miR-155 (Figure 7E).

## 3. Discussion

ZIKV is a recently emerged mosquito-transmitted flavivirus [3]. It is estimated that 80% of human infections are asymptomatic, and in the vast majority of symptomatic infections the disease is relatively mild and self-limiting [8]. In a small proportion of cases, the autoimmune-mediated Guillain–Barré syndrome can occur [9], but this can also occur with other mosquito-transmitted viruses such as West Nile virus [37], dengue virus [38] and chikungunya virus [39]. The most consequential ZIKV infections occur when a pregnant woman is infected in the first or second trimester of pregnancy. ZIKV can cross the placenta and cause numerous defects in the developing fetus [10]. One of the most severe defects is the destruction of neural progenitor cells in the developing brain, which results in microcephaly [40].

microRNAs are fundamental regulators of many biological processes, and they exert their effect at the post-translational level, through either repression of translation or degradation of cognate mRNAs [41]. In our screen of 50 candidate miRNAs, only 22 gave an amplification product on RNA derived from A549 cells (adenocarcinomic human alveolar basal epithelial cells), which is consistent with studies that have shown that while some miRNAs are ubiquitously expressed, others show expression in a limited number of cell types [42]. In addition, A549 cells are hypertriploid as well as containing numerous chromosomal rearrangements as well as chromosomal deletions [43] that are likely to affect miRNA expression. However, the results generated in A549 cells were largely recapitulated for selected miRNAs in NPCs derived from iPSCs. Markedly, all of the miRNAs investigated showed some degree of alteration of expression in response to ZIKV infection in A549 cells. The most common expression pattern was an early upregulation of the miRNA, followed by downregulation, followed by increased expression at the last time point examined. The reason for this pattern of miRNA expression remains unclear, although it is notable that a similar expression pattern was observed for miR-21 and miR-34a in ZIKV-infected NPCs. Somewhat remarkably, only 4 of the 22 miRNAs examined showed no differences in expression when comparing the two ZIKV isolates. Again, notably, differences in expression between the two ZIKV isolates were seen for miR-34a, miR-128 and miR-155 in ZIKV-infected NPCs. The two viruses (SV0010/15 and MU1-2017) differ by only nine amino acids [27], and as such the degree of discordant expression is somewhat surprising. It also suggests that comparing different studies using different isolates (and often different cell lines) must be undertaken cautiously.

From the 22 miRNAs evaluated, 4 (miR-21, miR-34a, miR-128 and miR-155) were selected for further investigation. miR-21 has been shown to promote DENV 2 replication in liver cells [23], and to induce cell proliferation during hepatitis B virus (HBV) infection [44], and overexpression of miR-21 also reduces oxidative stress and lipid accumulation [45,46]. miR-34a has multiple roles in cell cycle regulation, including lipid metabolism [47], and it has been reported to suppress the Wnt signaling pathway, resulting in the promotion of an antiviral response [22]. miR-128 shows antiviral effects towards human immunodeficiency virus 1 (HIV-1) [29] and human rhinovirus (HRV-1B) [30] replication. miR-155 inhibits dengue NS2B/NS3 protease activity through Bach1 and induces an antiviral interferon response resulting in the reduction of viral production [31]. miR-155 suppresses enterovirus 71 (EV71) by triggering the type I interferon (IFN-I) response [33] and indirectly induces the upregulation of luteolin, which inhibits respiratory syncytial virus (RSV) [32]. In ZIKV infection, all four selected miRNAs were antiviral in action, as downregulating miRNA expression through AMOs resulted in increased virus titer, and, conversely, adding an miRNA mimic resulted in reduced virus production on the second day after treatment for miRNA-34a in MU1-2017 and for both miR-34a and miR-128 in SV0010/15 infection.

Three potential targets of selected miRs, namely ALDOA (aldolase or fructose-bisphosphate A) and HSP70 (heat shock 70 kDa protein) as putative targets of miR-34a, and UBE2E2 (ubiquitin-conjugating enzyme E2 E2) as a potential target of miR-128 were selected for further investigation. ALDOA is a glycolytic enzyme that catalyzes the reversible conversion of fructose-1,6-bisphosphate to glyceraldehyde 3-phosphate and dihydroxyacetone phosphate at the fourth step of glycolysis [48], although this enzyme has recently been shown to play a pivotal role in the regulation of ribosomal biogenesis [49]. In addition, ALDOA has been shown to be a target of the ZIKV NS2B-NS3 protease, resulting in its proteolytic cleavage of ALDOA during ZIKV infection [50]. However, neither AMO-34a nor mimic-34a treatment resulted in changes in either RNA or protein that were consistent with the predicted alterations (increased for AMO treatment, decreased for mimic treatment), suggesting that while ALDOA is a putative target for miR-34a, it seems unlikely to be responsible for mediating the effects seen in ZIKV infection.

HSP70 is a member of the heat shock family of proteins, and primarily functions as a chaperone protein to help with the correct folding of proteins, and during cellular stress the protein can be highly upregulated to prevent unfolded protein aggregation and to protect cells against ER stress [51]. HSP70 has been shown to have dual roles in protection against apoptotic cell death, as well as to have pro-apoptotic functions under certain conditions [52]. HSP70 has been proposed to be required at multiple steps of ZIKV infection, replication and egress [53], and to be part of the JEV replication complex, where it acts to positively regulate genome regulation [54]. In addition, HSP70 has been proposed to act as a virus receptor for DENV [55] and JEV [56,57], although the DENV receptor activity is possibly cell type-specific [58]. In this study, HSP70 RNA was upregulated in response to AMO treatment on day 2 post-infection when infection was undertaken with MU1-2017, but no change was seen in RNA expression in ZIKV SV0010/15 infection. Mimic treatment resulted in strong downregulation of RNA for both MU1-2017 and SV0010/15 on both days of infection for both viruses, consistent with the expected result. In terms of protein expression, HSP70 was upregulated as a response to AMO-34a treatment in SV0010/15 infection, but not in MU1-2017 infection, and in response to mimic-34a treatment HSP70 expression was downregulated in MU1-2017 on day 2 post-infection, but was not downregulated in SV0010/15 infection on either day examined. Collectively, these results suggest that HSP70 plays a role in ZIKV infection through the action of miR-34a, but that the effect of the response can be modulated by strain type variation.

UBE2E2E is a ubiquitin-conjugating enzyme, acting as the middle enzyme of three in the process that attaches ubiquitin to cellular proteins [59]. There are few reports on the involvement of E2 ubiquitin-conjugating enzymes in virus infection, although UBE2J1 has been shown to mediate both DENV and ZIKV infection [60], while UBE2L3 has been shown to be a target of the chikungunya virus protease, and that restoring expression of UBE2L3 through transfection of a full-length clone reduced the level of infection and E protein expression [61]. However, treatment with AMO-128 produced almost no effect on either mRNA or protein expression of UBE2E2E, while mimic-128 showed effects at the RNA level, but none at the level of the protein. However, it should be noted that the effect of mimic-128 treatment on day 2 post-infection was discordant, as while SV0010/15 infection resulted in the expected downregulation of the UBE2E2E mRNA, infection with MU1-2017 resulted in an unexpected increase in RNA levels. Thus, again, it is possible that strain type differences play a role in the modulation of infection.

A number of studies have previously investigated the involvement of miRNAs in ZIKV infection [24,62,63,64,65,66,67,68,69,70,71,72,73,74,75,76,77]. These studies have variously identified the regulation of miR-302b, miR-302c, miR-194 and miR-30c in ZIKV-infected human NPCs in vitro [73], miR-146a in ZIKV-infected human microglial cells [75], hsa-miR-101-3p in ZIKV-infected human brain microvascular endothelial cells [67], mir-204 in human fetal neural stem cells as a consequence of ZIKV E protein expression [66], miR-7013-5p in ZIKV-infected fetal mouse neurons [62], miR-9 in ZIKV-infected mice [24] and miR-30e-3p, miR-30e-5p and miR-17-5p in ZIKV-infected astrocytes [70].

How extensive the remodeling of the miRNA circuitry is remains unclear. In one study on a neuronal cell line chronically infected with ZIKV, a total of 3192 miRNAs were evaluated, of which 389 were found to be upregulated more than 2-fold and 1291 were downregulated more than 2-fold [64]. miRNAs including hsa-mir-431-5p, hsa-mir-3687, hsa-mir-4655-5p, hsa-mir-6071, hsa-mir-762, hsa-mir-5787 and hsa-mir-6825-3p were significantly downregulated, while miRNAs including has-mir-4315, hsa-mir-5681b, hsa-mir-6511a-3p, hsa-mir-1264, hsa-mir-4418, hsa-mir-4497, hsa-mir-4485-3p, hsa-mir-4715-3p, hsa-mir-4433-3p, hsa-mir-4708-3p, hsa-mir-1973 and hsa-mir-564 were significantly upregulated [64]. However, a study in ZIKV-infected primary cultures of human fetal neural stem cells which looked at alterations of miRNA expression through global miRNA sequencing suggested that a more modest 14 miRNAs were upregulated and 11 miRNAs were downregulated [65]. In contrast, a study on Zika virus-infected neurons that examined 599 miRNAs showed a global downregulation of miRNAs with only a few upregulated miRNAs, and ZIKV-modulated miRNAs identified included miR-155, miR-203, miR-29a and miR-124-3p [63]. Examination of 754 miRNAs in a ZIKV-infected neuroblastoma cell line [68] identified seven downregulated miRNAs (miR-99a*, miR-126*, miR-190b, miR-361-3p, miR-522-3p, miR-299-5p and miR-1267) and one upregulated miRNA (miR145). In addition, four miRNAs were only found in ZIKV-infected cells (miR-148a, miR-342-5p, miR-598 and miR-708-3p) or mock cells (miR-208, miR-329, miR-432-5p, miR-488, miR-518b, miR-520g and miR-767-5p) and a further seven were found only in mock-infected cells (miR-208, miR-329, miR-432-5p, miR-488, miR-518b, miR-520g and miR-767-5p) [68]. Lastly, 35 differentially expressed miRNAs were identified by RNA-Seq analysis in ZIKV-infected A549 cells. Thus, unbiased global sequencing methodologies show wide variation in evaluating the degree of miRNA alterations in ZIKV infection. Our study used a candidate miRNA approach, and only half the candidates were expressed in A549 cells, but almost all of those candidates examined showed some degree of regulation as a consequence of ZIKV infection.

Perhaps the most striking result in our study was the high degree of strain type variation observed. Nearly all miRNAs examined showed statistically significant differences between the two strains used (MU1-2017 and SV0010/15). Several other studies have examined alterations in miRNA expression as a consequence of different ZIKV strains [69,71,72,74,76]. Strikingly, all of these studies compared the original African-lineage Ugandan isolate of ZIKV (MR766) to a more recent Asian-lineage ZIKV [69,71,72,74,76]. Four of the studies reported at least some strain type variation in miRNA expression between the two isolates [69,71,72,76], while one study used two isolates but apparently did not compare expression across the isolates [74].

Perhaps the major strength of our study is that the two ZIKV strains used (MU1-2017 and SV0010/15) are both recent isolates in Thailand of the Asian-lineage ZIKV, and differ by only nine amino acid coding changes [27]. These changes include mutations in the capsid protein (T106A), envelope protein (A599T), NS2A (A1204T and V1289A), NS3 (H2086Y) and NS5 (H2594Y, V2842I, Y2962H and P3162S). Remarkably, while extensive differences were seen at the level of the miRNA, the strain type-specific effects were also seen at the protein level of a selected putative target, HSP70. The results from this study and others [69,71,72,76] indicate that future studies on the modulation of miRNAs as a consequence of ZIKV infection must take into account strain type variation, as the results generated by a single strain will be of limited value in understanding the role of miRNAs in ZIKV infection.

## 4. Materials and Methods

### 4.1. Cell Culture

The whole larva *Aedes albopictus* mosquito cell line C6/36 (ATCC CRL-1660) was cultured in minimal essential medium (MEM; Gibco, Thermo Fisher Scientific, Waltham, MA, USA) supplemented with 10% heat-inactivated fetal bovine serum (FBS; Gibco) and 100 units/mL of penicillin/streptomycin (Merck, Darmstadt, Germany) at 28 °C. Cells were maintained in 182 cm^2^ tissue culture flasks (JETBIOFIL, Guangzhou, China) and subcultured twice per week. To subculture, medium was removed, and cells were washed with phosphate-buffered saline (PBS: 137 mM NaCl, 2.7 mM KCl, 4.29 mM Na_2_HPO_4_, 1.39 mM KH_2_PO_4_) after which cells were incubated with 5 mL 0.06% trypsin/EDTA (Gibco) at 37 °C for 5 min. Fresh complete medium was added to inactivate trypsin and then cells were pelleted by centrifugation at 1200 rpm for 3 min. The cell pellet was resuspended in 15 mL of complete media by pipetting through a 10 mL serological pipette 10–15 times. A total of 1–3 mL (1:15–1:5) of cell suspension then was subcultured to new tissue culture flasks. Fresh complete medium was added to a final volume of 30 mL. Cells were incubated at 28 °C.

The human lung carcinoma cell line A549 (ATCC CCL-185) and African green monkey kidney cell line Vero (ATCC CCL-81) were cultured in Dulbecco’s Modified Eagle Medium (DMEM; Gibco) supplemented with 10% and 5% FBS, respectively, at 37 °C with 5% CO_2_. Cells were subcultured as described above, except the concentration of trypsin was 0.25%.

The human-induced pluripotent stem cells (hiPSCs) (kindly provided by Associate Professor Methichit Wattanapanitch, Siriraj Center for Regenerative Medicine, Faculty of Medicine, Siriraj Hospital, Mahidol University, Thailand) [78] were cultured in mTeSR™1 media (STEMCELL Technologies, Vancouver, BC, Canada). Medium was replaced every two days for seven days before passage. On the day of passage, the culture medium was removed, and cells were washed with DMEM/F-12 (Hyclone Laboratories Inc., Logan, UT, USA). Subsequently, 1 mL of mTeSR™1 medium was added to the hiPSCs, and the hiPSCs colonies were dissociated to pieces of a size of approximately 1 × 1 mm. Medium containing hiPSCs colonies was transferred to a new microcentrifuge tube and the hiPSCs colonies were allowed to drop to the bottom of the tube for 5 min. After that, medium was removed and the hiPSCs colonies were transferred to a new Matrigel^®^ (Corning Inc., Corning, NY, USA) coated plate. hiPSCs were cultured in 2 mL mTESR™1 media at 37 °C with 5% CO_2_.

### 4.2. Neural Progenitor Cells Differentiation and Culture

Neural progenitor cells (NPCs) were derived from hiPSCs through the steps of neural induction, replating, rosette selection and neural rosette cell maintenance. Briefly, the hiPSCs were dissociated by incubation with ACCUTASE™ (STEMCELL Technologies) and placed in Aggrewell^TM^800 plates (STEMCELL Technologies) in a total of 2 mL of STEMdiff™ Neural Induction Medium containing ROCK inhibitor (Y-27632, STEMCELL Technologies). Cells were incubated at 37 °C with 5% CO_2_ for 4 days, with 75% medium replacement daily. On day five, embryoid bodies were collected and transferred to new Matrigel^®^ coated 6-well plates and the medium was fully replaced daily for 7 days. On day 12, neural rosettes were selected by STEMdiff™ Neural Rosette Selection Reagent (STEMCELL Technologies) and transferred to new Matrigel^®^ coated 6-well plates. Selected neural rosettes were cultured at 37 °C with 5% CO_2_ for 5–7 days, with the medium being replaced daily until the cells reached 100% confluence.

At 100% confluence of NPCs, the culture medium was removed, and cells were washed with 1 mL PBS. NPCs were dissociated and detached by pipetting 10 times followed by adding 5 mL DMEM/F-12. The NPCs suspension was then centrifuged, and cells were resuspended with NPC medium consisting of DMEM/F12, 0.5X N-2 (Gibco), 0.5X B-27 (Gibco), 1X Glutamax (Gibco), 1X penicillin/streptomycin and 20 ng/mL fibroblast growth factor 2 (FGF2, STEMCELL Technologies). Viable cells were counted using trypan blue and cells were plated onto a Matrigel^®^ coated plate or flask at the desired density (1.25 × 10^5^ cells/cm^2^). NPCs were incubated at 37 °C with 5% CO_2_, NPC medium were replaced every two days.

### 4.3. Virus Propagation

The Asian-lineage ZIKV used in this study was an isolate recovered from a case of CZS, ZIKV MU1-2017 [27], which is identical to the independently isolated [79] BKK02 (GenBank accession no. MF996804), and a Zika fever-associated isolate, ZIKV SV0010/15 (GenBank accession no. KX051562) [26], kindly provided by the Armed Forces Research Institute of Medical Sciences (AFRIMS), Thailand. The viruses were propagated in C6/36 cells. C6/36 cells were seeded into 182 cm^2^ cell culture flasks at a density allowing 80% confluence to be reached within 24 h. On the day of infection, cells were incubated with diluted virus in serum-free MEM medium at 28 °C for 2 h with constant agitation. MEM supplemented with 10% heated inactivated FBS was added to the infected cells, which were then incubated at 28 °C until the virus was harvested on the day in which the cells showed 80% cytopathic effects. On the day of collection, the supernatant containing the virus was harvested, followed by centrifugation at 1000× *g* for 5 min to remove detached cells. The viral supernatant was supplemented with 20% FBS and kept at −80 °C as stock for further experiments. The stock virus identities were confirmed by sequencing and viral titer was determined by plaque assay.

### 4.4. Plaque Assay

Vero cells were used to determine ZIKV titer. Briefly, cells were seeded onto 6-well cell culture plates under standard conditions at a density that allowed 100% confluence to be reached within 24 h. On the infection day, viruses were 10-fold serially diluted with BA-1 diluent (1× Medium 199, Earle’s Salts (Gibco), 0.05M Tris-HCl pH 7.6, 1% BSA fraction V, 0.075% sodium bicarbonate (NaHCO_3_), 100 units/mL penicillin/streptomycin) and added to the cells followed by incubation at 37 °C for 2 h with gentle agitation every 10 min. After that, cells were overlaid with 1× MEM containing 1.2% methyl cellulose (Sigma-Aldrich, St. Louis, MO, USA) supplemented with 2% FBS followed by incubation at 37 °C with 5% CO_2_ for 7 days. Then, cells were fixed with diluted 3.7% formaldehyde (Merck) in 1× PBS for 1 h followed by rinsing with water and staining with 1% crystal violet (Merck).

### 4.5. Virus Infection

A549 cells or NPCs were seeded onto cell culture plates at a density that allowed 50% confluence to be reached within 24 h. At the time of infection, media were aspirated immediately before inoculating the cells with 1 mL of ZIKV at the required multiplicity of infection (MOI) diluted in ice-cold serum-free DMEM medium for A549 cells and DMEM/F12 for NPCs. Cells were incubated with the virus at 37 °C for 2 h. For A549 cells, after virus incubation, pre-warmed DMEM supplemented with FBS was added to a final 10% FBS concentration. For NPCs, DMEM/F12 containing virus was replaced with pre-warmed NPCs medium. Cells were incubated at 37 °C with 5% CO_2_ until required.

### 4.6. Flow Cytometry

ZIKV-infected or mock-infected cells were harvested at the indicated time points. Cells were washed with PBS (137 mM sodium chloride, 2.7 mM potassium chloride, 4.3 mM disodium hydrogen phosphate, 1.4 mM potassium dihydrogen phosphate) and blocked with 10% goat serum (Gibco) on ice for 30 min. Then, cells were washed twice with PBS and were fixed with 4% paraformaldehyde (Merck) in PBS-IFA (154 mM sodium chloride, 50 mM disodium hydrogen phosphate, 50 mM sodium dihydrogen phosphate pH 7.4) for 20 min at room temperature in the dark. Cells were washed twice with 1% BSA in PBS-IFA and permeabilized with 0.2% Triton X-100 (Omnipur, Merck) for 10 min in the dark at room temperature followed by washing twice with 1% BSA/PBS-IFA. Then, cells were incubated overnight with a 1:3 dilution of mouse monoclonal anti-flavivirus envelope protein antibody (4G2, HB112; produced in-house) at 4 °C overnight. After that, cells were washed 3 times with 1% BSA/PBS-IFA and incubated with a 1:40 dilution of a FITC conjugated goat anti-mouse IgG antibody (KPL, Guilford, UK) for 1 h in the dark at room temperature. Subsequently, cells were washed 3 times with 1% BSA in PBS/IFA and diluted in PBS-IFA followed by analysis by flow cytometry on a BD FACSCalibur cytometer (BD, Franklin Lakes, NJ, USA) using CELLQuest^TM^ software, Version 3.3. All experiments were undertaken independently in triplicate.

### 4.7. Inhibition of miRNA Using Anti-miRNA Oligonucleotide (AMO) in ZIKV-Infected Cells

A549 cells were seeded into 12-well cell culture plates at a density that allowed 50% confluence to be reached within 24 h. At the time of infection, cells were infected with ZIKV at the required MOI for 2 h and subsequently incubated at 37 °C with 5% CO_2_ until required. At the time of transfection, an anti-miRNA oligonucleotide (AMO) (GenScript, Piscataway, NJ, USA) transfection reaction was prepared according to the manufacturer’s protocol. Briefly, 40 μL of 2 μM AMO stock was mixed with 40 μL reduced-serum media (Opti-MEM; Gibco). In parallel, 0.8 μL DharmaFECT I (Horizon Discovery, Cambridge, UK) was mixed with Opti-MEM to a final 80 μL and incubated for 5 min. Then, diluted AMO was added to the diluted DharmaFECT I and incubated for 20 min to form the AMO/DharmaFECT complex. After that, 640 μL complete DMEM medium was added to the AMO/DharmaFECT complex followed by adding it to the ZIKV-infected cells, which were incubated at 37 °C with 5% CO_2_ until required.

### 4.8. Mimic miRNA Transfection in ZIKV-Infected Cells

MISSION^®^ mimic miRNAs (Sigma-Aldrich, St. Louis, MO, USA) were reverse-transfected to A549 cells followed by ZIKV infection. Briefly, 10 μL of 2 μM mimic miRNA stock was mixed with 10 μL reduced-serum medium (Opti-MEM; Gibco). In parallel, 0.8 μL DharmaFECT I (Horizon Discovery, Cambridge, UK) was mixed with Opti-MEM to a final 20 μL and incubated for 5 min. Then the diluted mimic miRNA was mixed with the diluted DharmaFECT I in a 12-well cell culture plate followed by being incubated for 30 min to form the mimic miRNA/DharmaFECT complexes. Then, A549 cells were seeded into a 12-well cell culture plate to allow 50% confluence within 24 h. Subsequently, reverse-transfected cells were infected with ZIKV at the required MOI for 2 h and subsequently incubated at 37 °C with 5% CO_2_ until required.

### 4.9. RNA Extraction and Quantitative Reverse-Transcription PCR

RNA was extracted using Trizol reagent (Invitrogen, Thermo Fisher, Waltham, MA, USA) following the manufacturer’s protocol. RNA was diluted, aliquoted and stored at −80 °C. For miRNA detection, cDNA synthesis was then performed by mixing 90–150 ng of total RNA with specific primers (Supplemental Table S1) and pre-heating at 65 °C for 5 min followed by immediately placing samples on ice for 5 min. cDNA was synthesized using RevertAid Reverse Transcriptase (Thermo Fisher). The PCR conditions used followed Varkonyi-Gasic and colleagues [80]. Briefly, samples were incubated in a Veriti Thermo Cycler (Applied Biosystems, Thermo Fisher, Waltham, MA, USA) at 16 °C for 5 min followed by 60 cycles of 30 °C for 30 s, 45 °C for 30 s and 50 °C for 1 s followed by enzymatic activity inactivation at 85 °C for 5 min. Subsequently, cDNA was used to determine target miRNA expression by quantitative reverse-transcription PCR using KAPA SYBR^®^ FAST qPCR Master MIX (Merck). The cycle conditions were 95 °C for 5 min, followed by 40 cycles of denaturing at 95 °C for 15 s and annealing with extension at 50–60 °C for 20 s. Results were normalized against miRNA U_6_ [81,82,83].

For mRNA detection, cDNA was synthesized from 75 ng of total RNA mixed with random hexamers primer (Invitrogen) using RevertAid Reverse Transcriptase. The PCR conditions followed the manufacturer’s protocol. cDNA was used as a template to determine mRNA expression level using specific primers (Supplemental Table S2) and KAPA SYBR^®^ FAST qPCR Master MIX. The cycle conditions were 95 °C for 5 min, followed by 40 cycles of denaturing at 95 °C for 15 s, annealing at 55 °C for 20 s and extension at 72 °C for 20 s.

### 4.10. Protein Extraction and Western Blot Analysis

To detect protein expression, cells were collected and lysed with RIPA lysis buffer (1% NP-40, 0.5% sodium deoxycholate, 0.1% sodium dodecyl sulfate, 137 mM sodium chloride, 2.7 mM potassium chloride, 4.3 mM disodium hydrogen phosphate, 1.4 mM potassium dihydrogen phosphate). Subsequently, 20 μL of total cell lysate was mixed with 5 μL reducing protein sample buffer (62.5 mM Tris-HCL, 100 mM DTT, 4% sodium dodecyl sulfate, 20% glycerol, 0.015% bromophenol blue). Cell lysates were then boiled for 5 min followed by loading onto 10% sodium dodecyl sulfate polyacrylamide (SDS-PAGE) gels. After electrophoresis, proteins were transferred to Amersham Protra 0.2 μm nitrocellulose membranes (GE Healthcare Life Science, Marlborough, MA, USA) and membranes were blocked with 5% skim milk (Difco, Franklin Lakes, NJ, USA) in 0.05% (TBS-T 20 mM Tris-HCl, 140 mM NaCl, 0.05% Tween-20). Membranes were then incubated with a specific primary antibody followed by an appropriate secondary antibody conjugated with horseradish peroxidase (HRP) (Supplemental Table S3). The chemiluminescent signal was developed using Immobilon Forte Western HRP substrate (Merck) and detected using an AZURE c400 Gel Imaging System (AZURE Biosystems, Inc., Dublin, CA, USA). Protein band intensity was analyzed using the ImageJ program version 1.47 [84].

### 4.11. Immunofluorescence Assay

To detect cellular protein expression, cells were grown on glass coverslips and were fixed with 4% paraformaldehyde (Merck) in PBS-IFA (154 mM Sodium chloride, 50 mM disodium hydrogen phosphate, 50 mM sodium dihydrogen phosphate, pH 7.4). Cells were then permeabilized with 0.3% Triton^®^X-100 (Omnipur, Merck) and blocked with 2% fetal bovine serum (Gibco). Subsequently, cells were incubated with specific primary antibodies diluted in PBS-IFA followed by an appropriate secondary antibody conjugated with a fluorescence dye diluted in PBS-IFA as detailed in Supplemental Table S4. The fluorescent signal was observed under a Zeiss LSM 800 confocal laser scanning microscope (Zeiss Group, Oberkochen, Germany).

### 4.12. Statistical Analysis

All statistical analyses were performed using an independent *t*-test for quantitative RT-PCR and paired *t*-test for western blot analysis using Predictive Analytic Software 18 (PASW^®^ Statistics 18, IBM, Armonk, NY, USA) and GraphPad Prism program version 5.0 (GraphPad Software, San Diego, CA, USA). A *p*-value less than 0.05 was considered statistically significant.

## Figures and Tables

**Table 1:** Predicted target binding sites.

Predicted Target	Binding Site	Aggregate P_CT_ TargetScanHuman 7.1	Target Score miRDB
Position 242–249 of HSPA1B 3′ UTR hsa-miR-34a-5p	5′ CUUUAAAUGAAUCAACACUGCCA | | | | | | | 3′ UGUUGGUCGAUUCUGUGACGGU	0.87	91
Position 268–275 of ALDOA 3′ UTR hsa-miR-34a-5p	5′ UCACCCUUUCCGGCACACUGCCA | | | | | | | 3′ UGUUGGUCGAUUCUGUGACGGU	0.89	81
Position 2466–2472 of NOTCH2 3′ UTR hsa-miR-34a-5p	5′ UGAUGAGGAGGACAACACUGCCU | | | | | | | 3′ UGUUGGUCGAUUCUGUGACGGU	0.73	84
Position 92–99 of PHB 3′ UTR hsa-miR-128-3p	5′ UCCCACCCCAGAAAUCACUGUGA | | | | | | | 3′ UUUCUCUGGCCAAGUGACACU	0.85	95
Position 546–552 of UBE2E2 3′ UTR hsa-miR-128-3p	5′ AGCUUCAAUCAGAAUCACUGUGC | | | | | | | 3′ UUUCUCUGGCCAAGUGACACU	0.95	99
Position 1606–1613 of MSI2 3′ UTR hsa-miR-128-3p	5′ GUAUAAACAUCACUGCACUGUGA | | | | | | | 3′ UUUCUCUGGCCAAGUGACACU	0.86	97
Position 4831–4837 of MSI2 3′ UTR hsa-miR-128-3p	5′ UAAAACUUUCCCUAGCACUGUGG | | | | | | | 3′ UUUCUCUGGCCAAGUGACACU	0.86	97

## Data Availability

Data are contained within the article and Supplementary Materials.

## Supplementary Materials

The supporting information can be downloaded at: https://www.mdpi.com/article/10.3390/ijms242216216/s1.
