# Supplementary material for: Strain Variation Can Significantly Modulate the miRNA Response to Zika Virus Infection
Source: Int J Mol Sci. 2023 Nov 11;24(22):16216. doi: 10.3390/ijms242216216 (PMC10671159; doi:10.3390/ijms242216216)
Supplement: Supplementary file 1 [file ijms-24-16216-s001.zip › ijms-2657054-supplementary.pdf]

**Supplemental materials**

# Strain variation can significantly modulate the miRNA response to Zika virus infection

Suwipa Ramphan[1], Chanida Chumchanchira[2], Wannapa Sornjai[1], Thanathom Chailangkarn[3], Anan Jongkakaewwattana[3], Wanchai Assavalapsakul[4], Duncan R. Smith[1*]

[1]Institute of Molecular Biosciences, Mahidol University, Nakhon Pathom, 73170, Thailand

[2]Department of Biology, Faculty of Sciences, Chiang Mai University, Muang District, 50200, Thailand

[3]National Center for Genetic Engineering and Biotechnology, National Science and Technology Development Agency, Pathum Thani, 12120, Thailand

[4]Department of microbiology, Faculty of Sciences, Chulalongkorn University, Bangkok, 10330, Thailand.

*Correspondence to: Duncan R. Smith, Molecular Pathology Laboratory, Institute of Molecular Biosciences, Mahidol University, Salaya Campus, 25/25 Phuttamonthon Sai 4, Salaya, Nakhon Pathom, Thailand 73170; Phone: 66(0) 2441-9003 to 7. Fax: 66 (0) 2441-1013. E-mail: duncan_r_smith@hotmail.com, duncan.smi@mahidol.ac.th

**Supplemental Table S1. Specific primers for miRNA detection**

| Primer name | Sequence (5'-3') |
|---|---|
| $U_6$_RT | TATGGAACGCTTC |
| $U_6$_F | CTCGCTTCGGCAGCACA |
| $U_6$_R | ACGCTTCACGAATTTGCGTGTC |
| universal_R | GTGCAGGGTCCGAGGT |
| hsa-miR-10b-5p_RT | GTCGTATCCAGTGCAGGGTCCGAGGTATTCGCACTGGATACGACCACAAA |
| hsa-miR-10b-5p_F | AAAAATACCCTGTAGAACCGAA |
| hsa-miR-15b-5p_RT | GTCGTATCCAGTGCAGGGTCCGAGGTATTCGCACTGGATACGACTGTAAA |
| hsa-miR-15b-5p_F | CACTATAGCAGCACATCATGG |
| hsa-miR-16-3p_RT | GTCGTATCCAGTGCAGGGTCCGAGGTATTCGCACTGGATACGACTCAGCA |
| hsa-miR-16-3p_F | CTGACCCAGTATTAACTGTGC |
| hsa-miR-106b-5p_RT | GTCGTATCCAGTGCAGGGTCCGAGGTATTCGCACTGGATACGACATCTGC |
| hsa-miR-106b-5p_F | GGGGCTAAAGTGCTGACAGT |
| hsa-miR-125a-5p_RT | GTCGTATCCAGTGCAGGGTCCGAGGTATTCGCACTGGATACGACTCACAG |
| hsa-miR-125a-5p_F | CTTTTTCCCTGAGACCCTTTAAC |
| hsa-miR-128-3p_RT | GTCGTATCCAGTGCAGGGTCCGAGGTATTCGCACTGGATACGACAAAGAGAC |
| hsa-miR-128-3p_F | TCAGGTGTCACAGTGAACCG |
| hsa-miR-155-5p_RT | GTCGTATCCAGTGCAGGGTCCGAGGTATTCGCACTGGATACGACAACCCC |
| hsa-miR-155-5p_F | CGAGTTTAATGCTAATCGTGATA |
| hsa-miR-192-5p_RT | GTCGTATCCAGTGCAGGGTCCGAGGTATTCGCACTGGATACGACGGCTGT |
| hsa-miR-192-5p_F | CGTGGCTGACCTATGAATTG |
| hsa-miR-21-5p_RT | GTCGTATCCAGTGCAGGGTCCGAGGTATTCGCACTGGATACGACTCAACA |
| hsa-miR-21-5p_F | GCCCGTAGCTTATCAGACTGA |
| hsa-miR-215-5p_RT | GTCGTATCCAGTGCAGGGTCCGAGGTATTCGCACTGGATACGACGTCTGT |
| hsa-miR-215-5p_F | GGGCGTATGACCTATGAATTG |
| hsa-miR-218-5p_RT | GTCGTATCCAGTGCAGGGTCCGAGGTATTCGCACTGGATACGACACATGG |
| hsa-miR-218-5p_F | CCGTTCTTGTGCTTGATCTAA |
| hsa-miR-23b-3p_RT | GTCGTATCCAGTGCAGGGTCCGAGGTATTCGCACTGGATACGACGGTAAT |
| hsa-miR-23b-3p_F | GGACGATCACATTGCCAGGG |

| | |
|---|---|
| hsa-miR-27a-3p_RT | GTCGTATCCAGTGCAGGGTCCGAGGTATTCGCACTGGATACGACGCGGAA |
| hsa-miR-27a-3p_F | GGGGTTTCACAGTGGCTAAG |
| hsa-miR-29b-3p_RT | GTCGTATCCAGTGCAGGGTCCGAGGTATTCGCACTGGATACGACAACACT |
| hsa-miR-29b-3p_F | CTGATAGCACCATTTGAAATC |
| hsa-miR-30a-3p_RT | GTCGTATCCAGTGCAGGGTCCGAGGTATTCGCACTGGATACGACGCTGCA |
| hsa-miR-30a-3p_F | CTTGTCTTTCAGTCGGATGTT |
| hsa-miR-30b-5p_RT | GTCGTATCCAGTGCAGGGTCCGAGGTATTCGCACTGGATACGACAGCTGA |
| hsa-miR-30b-5p_F | CTCCTTGTAAACATCCTACAC |
| hsa-miR-30e-5p_RT | GTCGTATCCAGTGCAGGGTCCGAGGTATTCGCACTGGATACGACCTTCCA |
| hsa-miR-30e-5p_F | CGTTCTGTAAACATCCTTGAC |
| hsa-miR-34a-5p_RT | GTCGTATCCAGTGCAGGGTCCGAGGTATTCGCACTGGATACGACACAACC |
| hsa-miR-34a-5p_F | GCTATTGGCAGTGTCTTAGCT |
| hsa-miR-424-5p_RT | GTCGTATCCAGTGCAGGGTCCGAGGTATTCGCACTGGATACGACTTCAAAAC |
| hsa-miR-424-5p_F | CTATAGCAGCAGCAATTCAT |
| hsa-miR-497-5p_RT | GTCGTATCCAGTGCAGGGTCCGAGGTATTCGCACTGGATACGACACAAAC |
| hsa-miR-497-5p_F | TATCACAGCAGCACACTGTG |
| hsa-miR-532-5p_RT | GTCGTATCCAGTGCAGGGTCCGAGGTATTCGCACTGGATACGACACGGTCCTA |
| hsa-miR-532-5p_F | ATCATGACATGCCTTGAGTG |
| Let-7a-5p_RT | GTCGTATCCAGTGCAGGGTCCGAGGTATTCGCACTGGATACGACAACTAT |
| Let-7a-5p_F | GCTAGTGAGGTAGTAGGTTGT |

**Supplemental Table S2 Specific primers for mRNA detection**

| Gene name | Primer name | Sequence (5'-3') |
|---|---|---|
| Aldolase A, fructose-bisphosphate | ALDOA_F | GGACAAATGGCGAGACTAC |
| | ALDOA_R | CTGGCAGATACTGGCATAAC |
| Heat shock 70kDa protein 1B | HSPA1B_F | ATCAGCCAGAACAAGCGAG |
| | HSPA1B_R | ACGTGTAGAAGTCGATGCC |
| Notch 2 | NOTCH2_F | GCAAGTCTCAGAAGCTAACC |
| | NOTCH2_R | ACATCTAACACATCCACCTCC |
| Musashi RNA-binding protein 2 | MSI2_F | ATCCCACTACGAAACGCTCC |
| | MSI2_R | TTCGCAGATAACCCGCCTAC |
| Prohibitin | PHB1_F | GCTGTCATCTTTGACCGATTCC |
| | PHB1_R | GCACACGCTCATCATAGTCC |
| Ubiquitin-conjugating enzyme E2E 2 | UBE2E2_F | TGGACCCAAAGGAGACAAC |
| | UBE2E2_R | AGCAGATGGAGAGGAGAAC |
| Glyceraldehyde-3-Phosphate Dehydrogenase | GAPDH_F | AGCCACATCGCTCAGACAC |
| | GAPDH_R | GCCCAATACGACCAAATCC |

**Supplemental Table S3 Antibodies list for western blot**

| Antibody | Dilution | Cat. No. | Company |
|---|---|---|---|
| Aldolase A | 1:5,000 | 3188 | Cell Signaling Technology, Danvers, MA |
| Heat shock protein 70 | 1:10,000 | SC-1060-R | Santa Cruz Biotechnology, Inc.,Dallas, Tx |
| Prohibitin (E-5) | 1:1,000 | SC-377037 | Santa Cruz Biotechnology |
| Actin-HRP | 1:15,000 | SC-47778 HRP | Santa Cruz Biotechnology |
| GAPDH | 1:10,000 | SC-32233 | Santa Cruz Biotechnology |
| Goat anti rabbit IgG-HRP | 1:8,000 | 31460 | Invitrogen, Waltham, MA |
| Rabbit anti mouse IgG-HRP | 1:8,000 | A5420 | Sigma-Aldrich, St. Louis, MO |

**Supplemental Table S4 Antibodies list for immunofluorescence assay**

| Antibody | Dilution | Cat. No. | Company |
|---|---|---|---|
| Rabbit anti human PAX-6 | 1:50 | 60094 | STEMCELL Technologies, Vancouver, Canada |
| Mouse anti human Nestin (10C2) | 1:500 | 60091 | STEMCELL Technologies, Vancouver, Canada |
| Goat anti SOX1 | 1:50 | AF3369 | R&D systems, Inc., Minneapolis, MN |
| Rabbit anti Musachi1 (EP1302) | 1:500 | AB52865 | Abcam, Cambridge, UK |
| Mouse anti GFAP | 1:25 | MA512023 | Invitrogen |
| Mouse anti Oct-3/4 (C-10) | 1:50 | SC-5279 | Santa Cruz Biotechnology |
| Goat anti human IgG-647 | 1:100 | A21445 | Invitrogen |
| Donkey anti mouse IgG-488 | 1:100 | A21202 | Invitrogen |
| Donkey anti Goat IgG-568 | 1:100 | A11057 | Invitrogen |
| Donkey anti rabbit IgG-647 | 1:100 | A31573 | Invitrogen |

**Supplemental Table S5. miRNA detection in A549 cells by PCR**

| miRNA | RT-PCR | miRNA | RT-PCR | miRNA | RT-PCR |
|---|---|---|---|---|---|
| 21 | ✓ | 128 | ✓ | 497 | ✓ |
| 10b | ✓ | 142 | × | 449a | × |
| 15a | × | 146a | × | 449b | × |
| 15b | ✓ | 148 | × | 517a | × |
| 16 | ✓ | 149 | × | 517c | × |
| 20a | × | 150 | × | 520a | × |
| 23b | ✓ | 155 | ✓ | 520c | × |
| 27a | ✓ | 192 | ✓ | 532 | ✓ |
| 29b | ✓ | 204 | × | 574 | × |
| 30a | ✓ | 215 | ✓ | 744 | × |
| 30b | ✓ | 218 | ✓ | 644a | × |
| 30e-3p | × | 221 | × | Let-7a | ✓ |
| 30e-5p | ✓ | 302b | × | Let-7c | × |
| 34a | ✓ | 302c | × | aae-2940 | × |
| 34c | × | 302d | × | | |
| 106b | ✓ | 339 | × | | |
| 124 | × | 375a | × | | |
| 125a | ✓ | 424 | ✓ | | |

✓ PCR products obtained, × no PCR product obtained.

**Supplemental Table S6. miRNA detection in NPCs by PCR**

| miRNA | RT-PCR | miRNA | RT-PCR | miRNA | RT_PCR |
|---|---|---|---|---|---|
| 10b | × | 30b | ✓ | 215 | × |
| 15b | ✓ | 30e-5p | ✓ | 218 | ✓ |
| 16 | × | 34a | ✓ | 424 | ✓ |
| 21 | ✓ | 106b | ✓ | 497 | × |
| 23b | ✓ | 125a | ✓ | 532 | × |
| 27a | × | 128 | ✓ | Let-7a | × |
| 29b | × | 155 | ✓ | | |
| 30a | × | 192 | × | | |

✓ PCR products obtained, × no PCR product obtained.

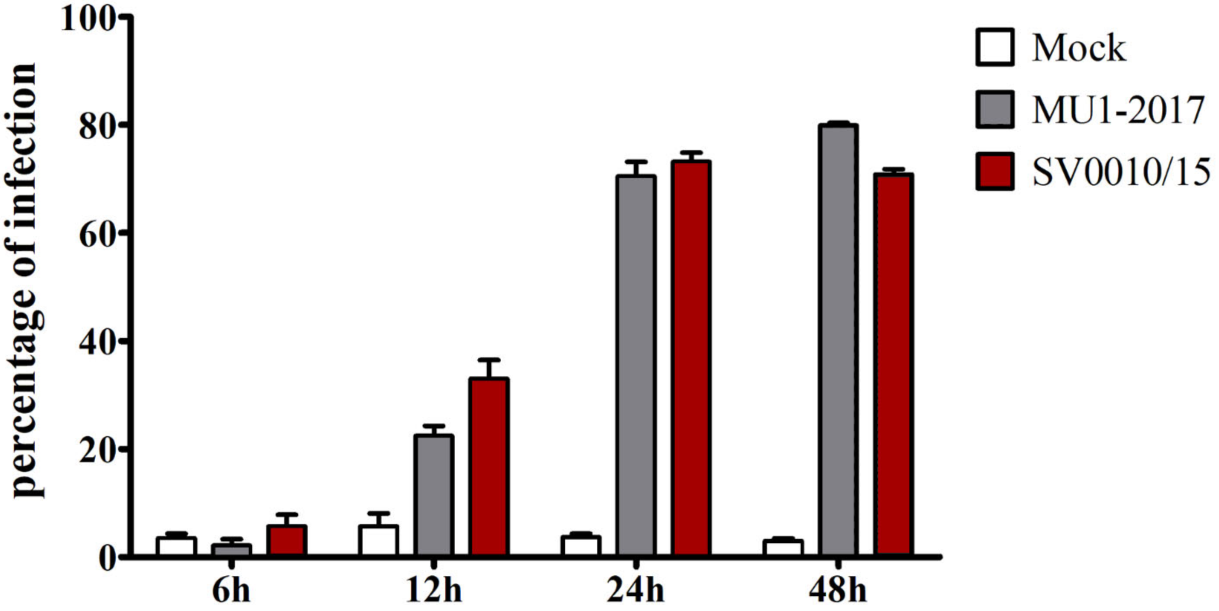


**Supplemental Figure S1.** Percentage of each ZIKV infection in A549 cells at 6, 12, 24 and 48 hours.

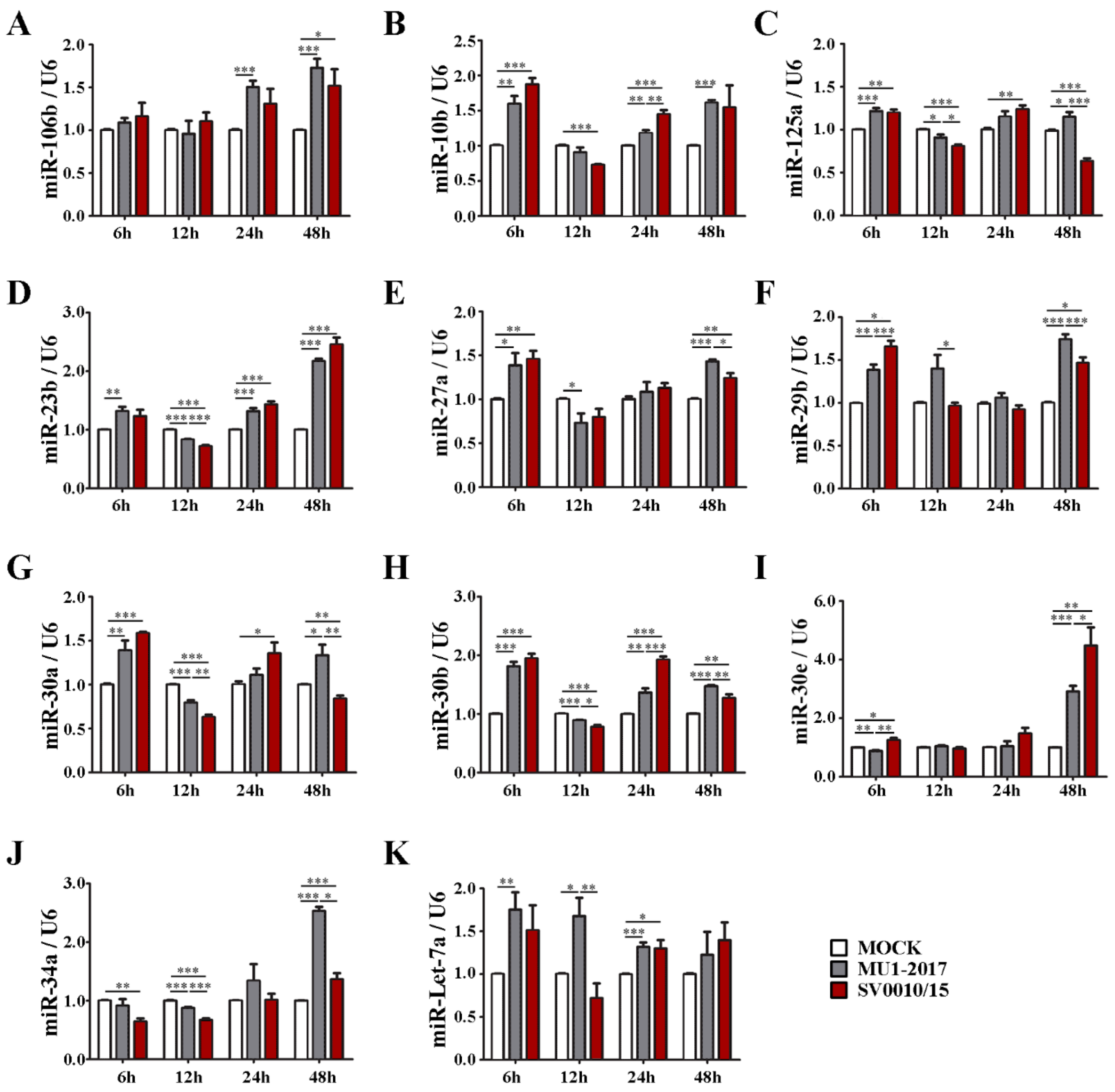


**Supplemental Figure S2.** miRNA (miR-106b, miR-10b, miR-125a, miR-23b, miR-27a, miR-29b, miR-30a, miR-30b, miR-30e, miR-34a, miR-Let-7a) expression levels after infection with one of two strains of ZIKV. A549 cells were infected with ZIKV MU1-2017 or ZIKV SV0010/15 at MOI 2 for 6, 12, 24 and 48 hours. (A-K) Level expression of each miRNA were quantified by RT-qPCR using specific primers with a 2-ΔΔCT calculation method and a small nuclear RNA, U6 was used as an internal control. All experiments were undertaken independently as biological triplicates, error bars represent standard error of mean. p value * < 0.05, ** < 0.01 and *** < 0.001.

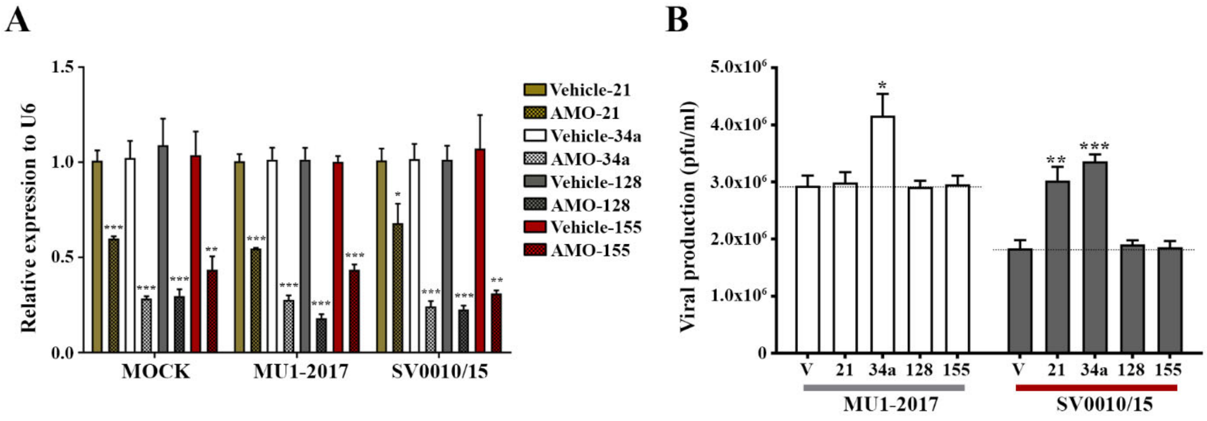


**Supplemental Figure S3**. Inhibition of miRNA in ZIKV infected cells. A549 cells were transfected with each AMO (or with vehicle as a control) for 24 hours before infection with ZIKV MU1-2017 or ZIKV SV0010/15. On day 2 post-infection cells were collected and RNA extracted to determine the miRNA expression levels and the supernatant was collected to determine viral production. Relative miRNA expression level in AMO treated cells compared to the vehicle control was determined by RT-qPCR with U6 used as an internal control (A). Viral production after AMOs treatment was determined by plaque assay (B). Experiments were undertaken independently in biological triplicate with technical duplicate plaque assay and technical triplicate RT-qPCR, error bars represent standard error of mean. p value * < 0.05, ** < 0.01 and *** < 0.001.

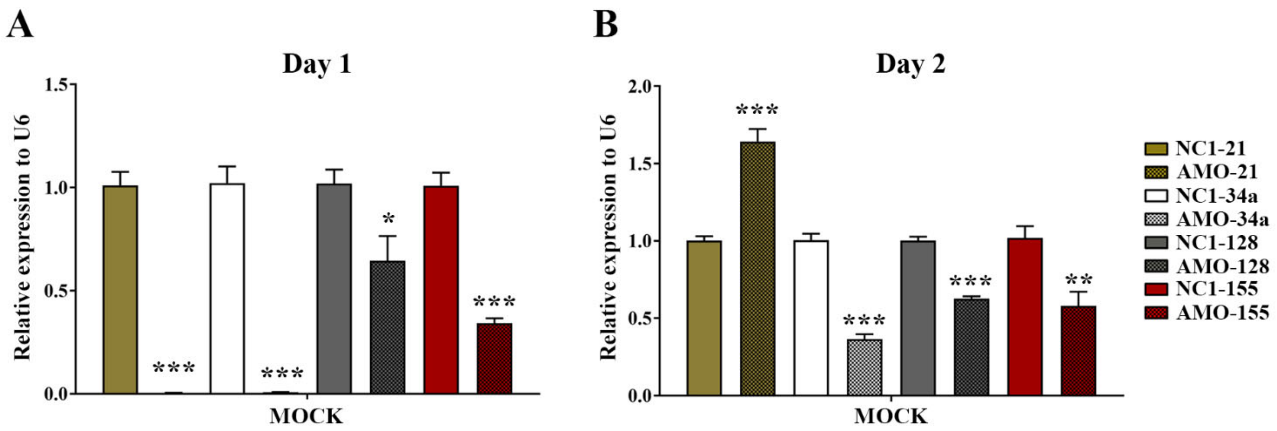


**Supplemental Figure S4.** Inhibition of miRNA in mock infected A549 cells. A549 cells were mock infected followed by transfection with each AMO. AMO-NC1 was used as a scrambled control. On days 1 to 2 post-infection cells were collected and RNA extracted to determine the miRNA expression levels. Relative miRNA expression level in AMO treated cells compared to the scramble control was determined by RT-qPCR with U6 used as an internal control (A, B). Experiments were undertaken independently in biological triplicate with technical triplicate RT-qPCR, error bars represent standard error of mean. p value * < 0.05, ** < 0.01 and *** < 0.001.

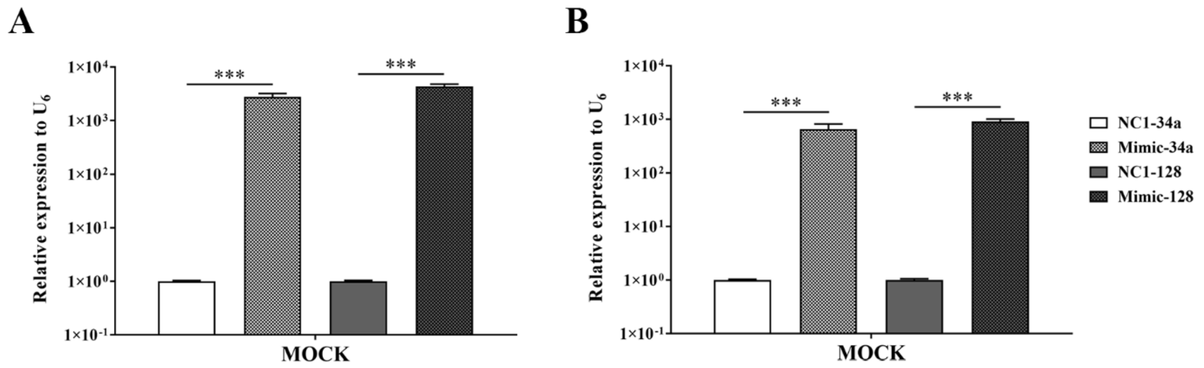


**Supplemental Figure S5**. Overexpression of miRNAs in mock infected A549 cells. Mimic miRNAs (mimic-34a and mimic-128) were reverse transfected into cells followed by mock infection. Mimic-NC1 was used as a scrambled control. RNA from cells was collected to determine the miRNAs expression level on day 1 (A), and 2 (B) post infection. Relative miRNA expression level in mimic miRNA treated cells compared to the scramble control, with U6 was used as an internal control. Experiments were undertaken independently as biological triplicates with technical triplicate RT-qPCR, error bars represent standard error of mean. p value * < 0.05, ** < 0.01 and *** < 0.001.

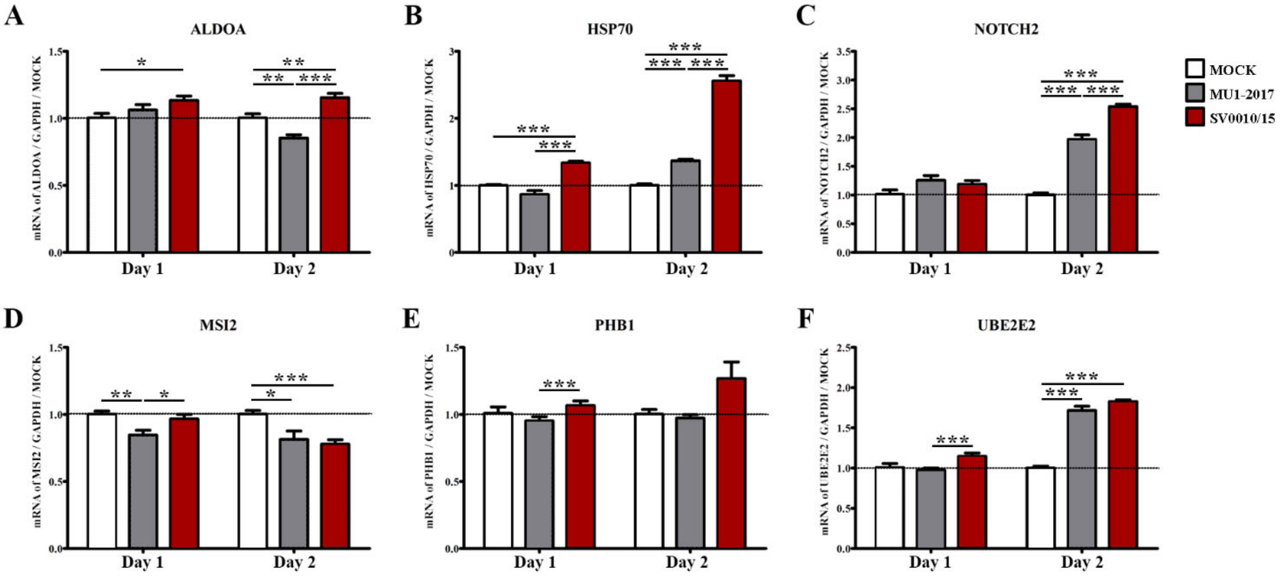


**Supplemental Figure S6.** mRNA expression levels of normal infection. (A) ALDOA, (B) HSP70, (C) NOTCH2, (D) MSI2, (E) PHB1 and (F) UBE2E2. Target mRNA normalized to GAPDH and normalized again to mock. *P<0.05, **P<0.01, ***P< 0.001

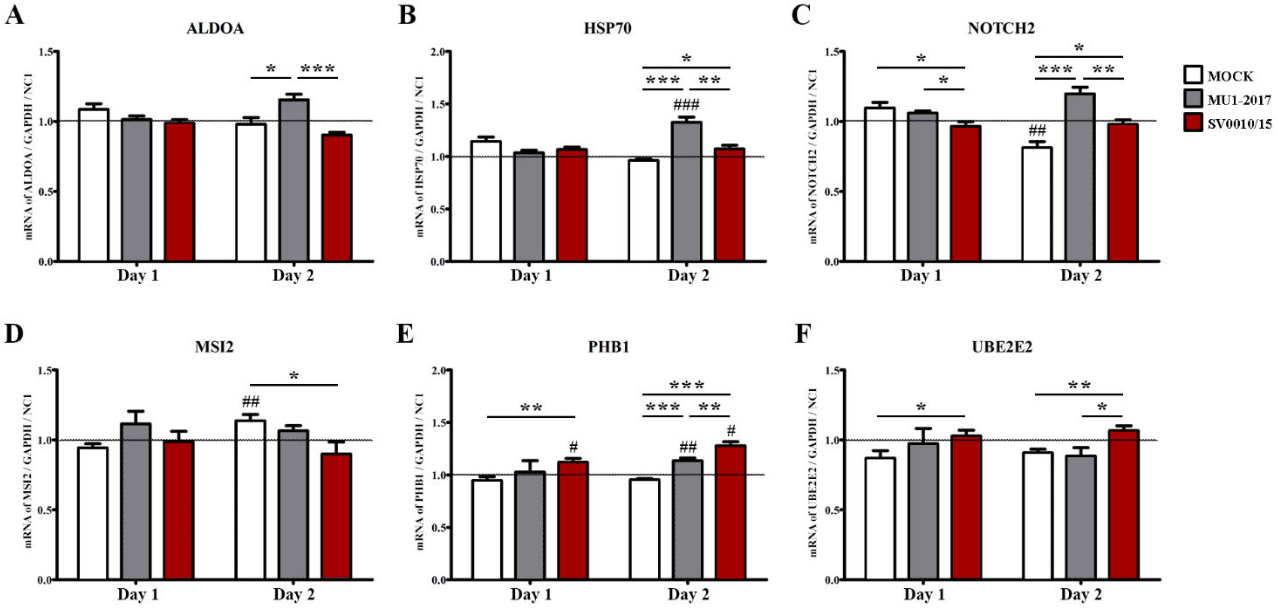


**Supplemental Figure S7.** mRNA expression levels of mock-infected or ZIKV-infected followed by AMO treated cells. AMO-34a treated (A) ALDOA, (B) HSP70 and (C) NOTCH2. AMO-128 treated (D) MSI2, (E) PHB1 and (F) UBE2E2. Target mRNA normalized to GAPDH and normalized again to NC1 control. *P<0.05, **P<0.01, ***P< 0.001 when comparing between groups, #P<0.05, ##P<0.01, ###P< 0.001 when comparing between sample and scrambled control.

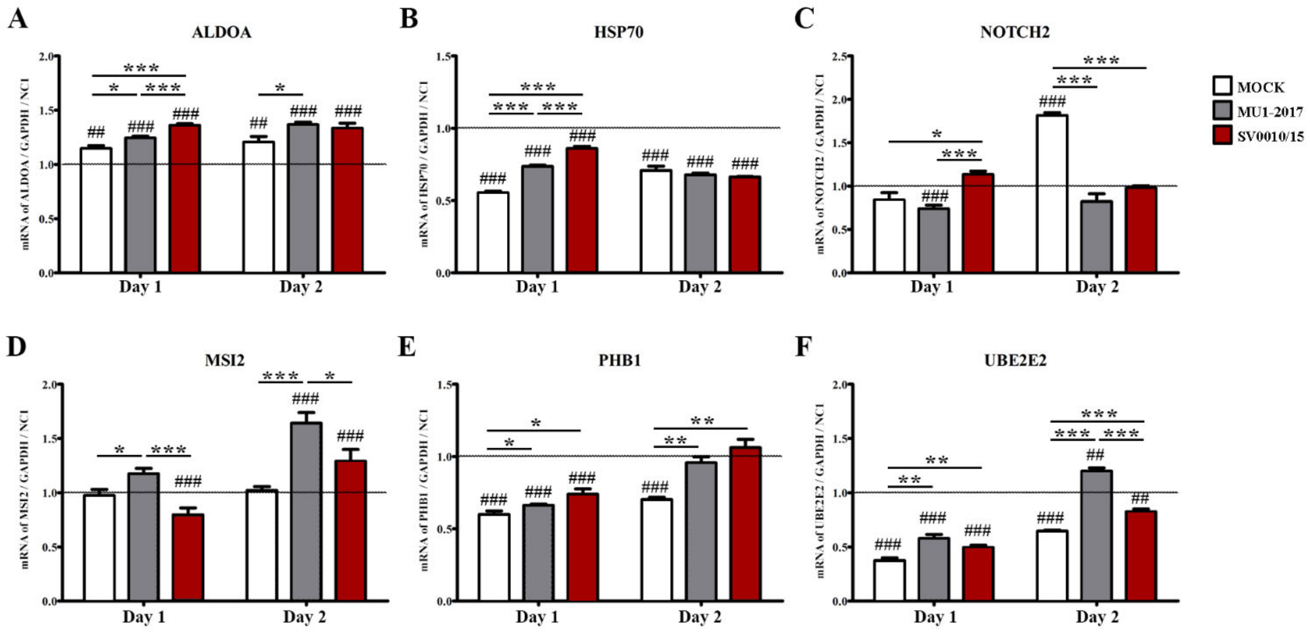


**Supplemental Figure S8.** mRNA expression levels of mimic treated cells followed by mock-infected or ZIKV-infected. mimic-34a treated (A) ALDOA, (B) HSP70 and (C) NOTCH2. mimic-128 treated (D) MSI2, (E) PHB1 and (F) UBE2E2. Target mRNA normalized to GAPDH and normalized again to NC1 control. *P<0.05, **P<0.01, ***P< 0.001 when comparing between groups, #P<0.05, ##P<0.01, ###P< 0.001 when comparing between sample and scrambled control.

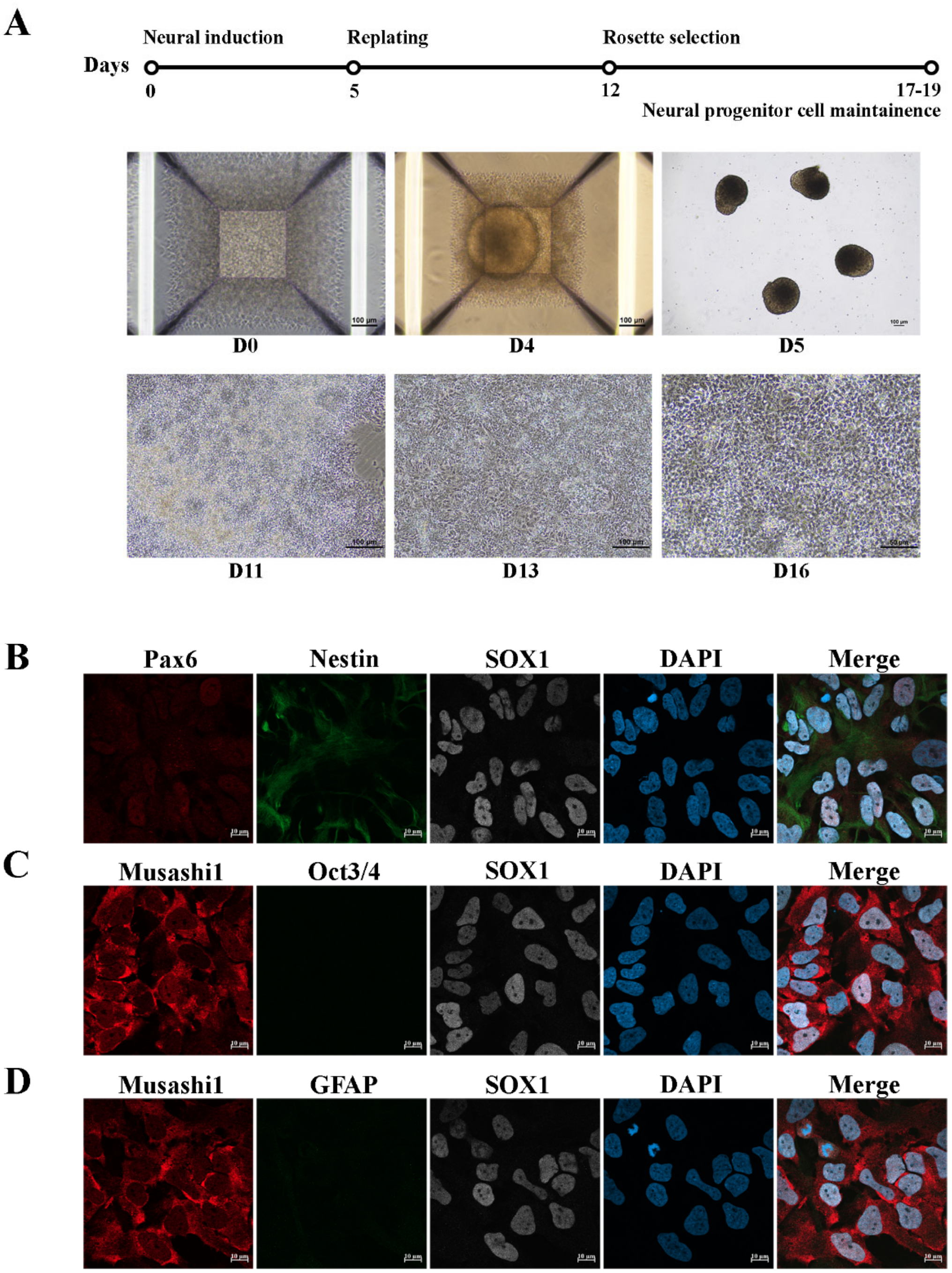


**Supplemental Figure S9.** NPCs differentiation stage and NPC marker staining. (A) Stage of NPCs differentiation and marker staining (B) nestin (green), PAX6 (red) and DAPI (blue). (C) SOX1 (white), nestin (green), Musashi 1 (red) and DAPI (blue). (D) SOX1 (white), glial fibrillary acidic protein, GFAP (green), Musashi 1 (red) and DAPI (blue).

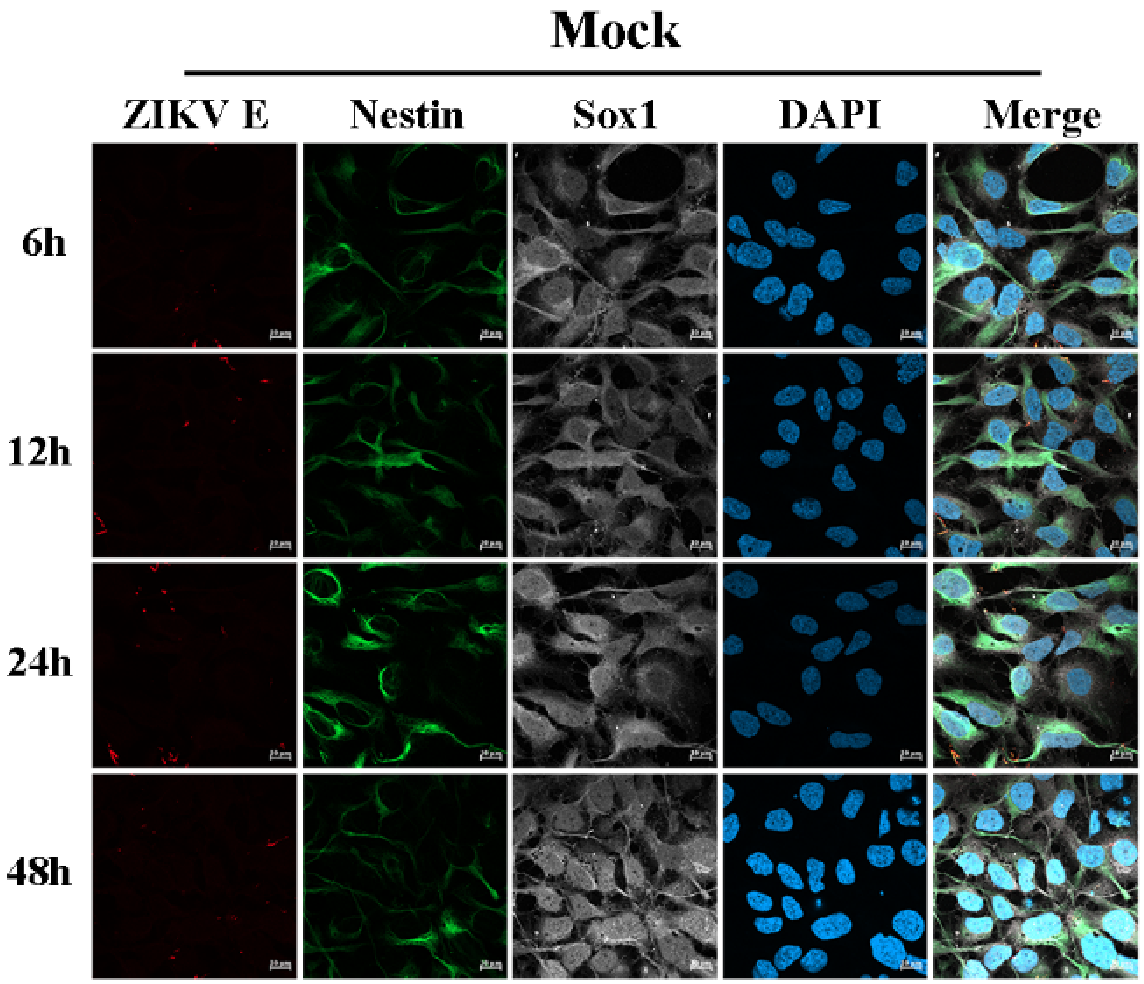


**Supplemental Figure S10.** Mock infection at 6, 12, 24 and 48 hours . ZIKV E protein shows in red, NPCs marker are nestin (green) and sox1 (white), nucleus was stained with DAPI (blue).
